# Functional genomic screens uncover FERMT2 as a critical regulator of YAP/TAZ-driven tumorigenicity

**DOI:** 10.1038/s41418-026-01694-w

**Published:** 2026-03-06

**Authors:** Arianna Chiesa, Vittoria Poli, Ottavio Croci, Francesca Biagioni, Patricio Fuentes, Mattia Marenda, Ambra Dondi, Simona Rodighiero, Marco Filipuzzi, Silvia Sberna, Matteo Marzi, Francesco Nicassio, Johannes Zuber, Stefano Campaner

**Affiliations:** 1 Center for Genomic Science of IIT, CGS@SEMM (Istituto Italiano di Tecnologia at the European School of Molecular Medicine), Fondazione Istituto Italiano di Tecnologia (IIT), Milan, Italy; 2 Imaging Unit, Department of Experimental Oncology, European Institute of Oncology (IEO), IRCCS, Milan, Italy; 3 Research Institute of Molecular Pathology (IMP), Vienna BioCenter (VBC), Vienna, Austria; 4 Medical University of Vienna, Vienna BioCenter (VBC), Vienna, Austria; 5 Department of Molecular Medicine, University of Padua, Padua, Italy

**Keywords:** Integrins, Oncogenes

## Abstract

YAP and TAZ are transcriptional regulators essential for mechanotransduction, development, and tissue homeostasis, whose dysregulation is implicated in multiple diseases, including cancer. To identify key regulators of YAP/TAZ signaling required for breast cancer cell fitness, we performed CRISPR/Cas9-based loss-of-function genetic screens both in vitro and in vivo. A custom sgRNA library targeting 216 candidate YAP/TAZ modulators was screened across three breast cancer cell lines. Among these, FERMT2, a component of the integrin signaling pathway, consistently emerged as a strong drop-out hit, highlighting its essential role in sustaining YAP/TAZ-dependent fitness. Bioinformatic analysis of large-scale cancer datasets further revealed genetic co-dependency between FERMT2, YAP, and TAZ, particularly in tumors with high YAP/TAZ expression. Functional validation through FERMT2 knockout and silencing demonstrated its requirement for proliferation, anchorage-independent growth, and tumorigenicity in triple-negative breast cancer cells. FERMT2 loss impaired YAP/TAZ nuclear accumulation, reduced the expression of YAP/TAZ target genes, and decreased phosphorylation at key tyrosine residues. Mechanistically, FERMT2 regulates YAP/TAZ independently of the canonical Hippo pathway through integrin-mediated activation of FAK. Consistent with this, glucocorticoid-driven FAK activation restored YAP/TAZ signaling in FERMT2-depleted cells. Partial epistasis analyses also indicate that FERMT2 modulates actin-dependent regulation of YAP/TAZ. Together, these findings identify FERMT2 as a pivotal upstream regulator of YAP/TAZ via FAK signaling, demonstrate that YAP/TAZ are principal effectors of integrin activity, and suggest that FERMT2 may represent a selective vulnerability in cancers with elevated YAP/TAZ signaling.

**Figure:**
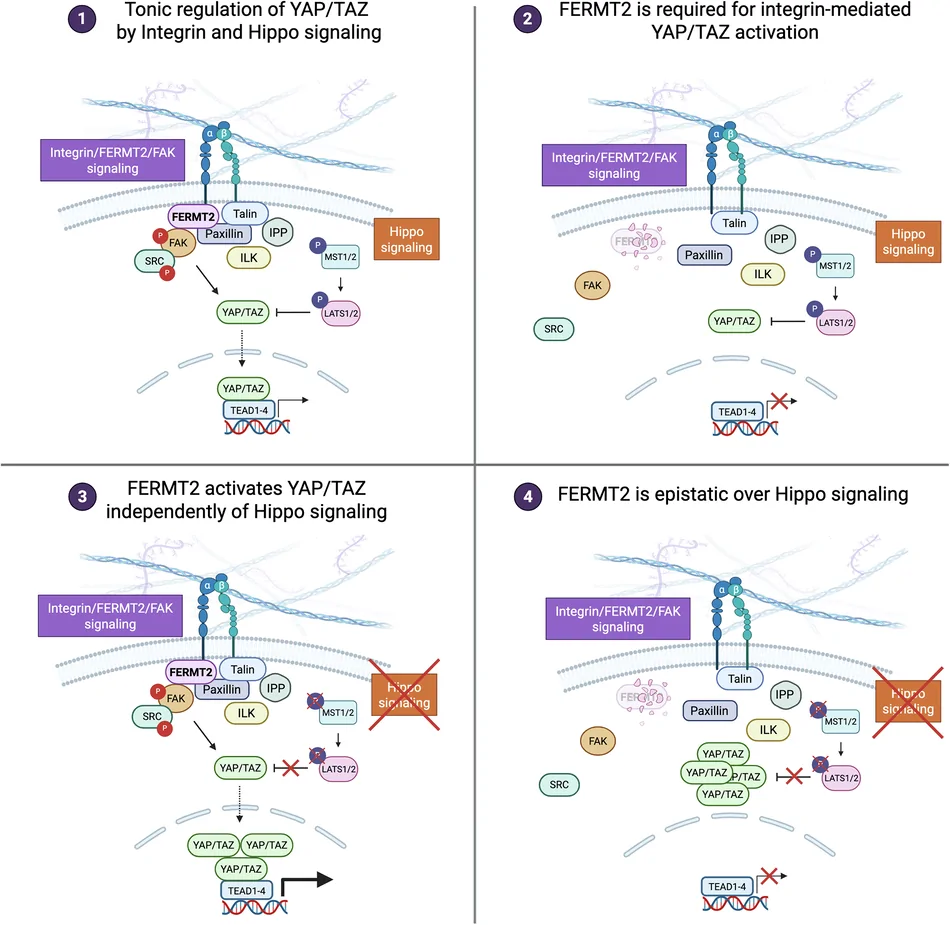

## Introduction

The YAP (Yes-associated protein) and TAZ (WWTR1, transcriptional coactivator with PDZ-binding motif) proteins are key transcriptional regulators that play crucial roles in mechanotransduction, organ development, tissue homeostasis, and regeneration [1]. They act as nuclear coactivators of TEAD transcription factors [2, 3], controlling the expression of genes involved in cell proliferation, differentiation, and survival [2]. Under physiological conditions, YAP/TAZ activity is tightly controlled to maintain balanced tissue growth and repair. However, their dysregulation contributes to various pathological conditions as cancer progression, fibrosis, and developmental disorders [4–6]. The regulation of YAP/TAZ is influenced by multiple upstream pathways, including the Hippo signaling pathway [7], adhesion and cytoskeletal tension [8–12], the WNT/β catenin pathway [13, 14], G-protein coupled receptor signaling [15, 16] and integrin-mediated focal adhesion kinase (FAK) signaling [7, 16]. These pathways modulate YAP/TAZ intracellular localization and activity in response to biochemical and mechanical cues.

The Hippo signaling pathway is a central regulator of YAP/TAZ activity. It consists of a kinase cascade that includes MST1/2 (mammalian Ste20-like kinases) and LATS1/2 (large tumor suppressor kinases), which phosphorylate YAP and TAZ, leading to their cytoplasmic retention and degradation. When Hippo signaling is active, phosphorylated YAP/TAZ bind to 14-3-3 proteins in the cytoplasm, preventing their nuclear translocation and transcriptional activity. Conversely, when Hippo signaling is suppressed, YAP/TAZ accumulate in the nucleus, interact with TEAD transcription factors, and promote gene expression programs that drive cell proliferation and survival.

Beyond Hippo signaling, actin polymerization, Rho GTPase activity, and mechanical stretching of the cytoskeleton influence YAP/TAZ nuclear localization and transcriptional activity [8–10]. Under conditions of increased cytoskeletal tension, such as during cell spreading or matrix stiffening, YAP/TAZ translocate into the nucleus and become transcriptionally active. In contrast, disruption of actin polymerization or inhibition of Rho GTPase signaling suppresses YAP/TAZ activity. This regulation allows cells to respond dynamically to mechanical changes in their microenvironment, integrating physical forces with gene expression programs that dictate cell fate decisions. Thus, the cytoskeleton serves as a critical intermediary between extracellular mechanical cues and intracellular YAP/TAZ signaling.

An additional regulatory mechanism involves integrin-mediated FAK signaling [12, 16, 17], which connects extracellular matrix (ECM) stiffness and cell adhesion to YAP/TAZ regulation. Integrins are transmembrane receptors that play a crucial role in cell adhesion, migration, and signal transduction [18]. These heterodimeric proteins, composed of α and β subunits, mediate cell-ECM interactions, thereby influencing various cellular processes such as proliferation, differentiation, and survival [19–21]. Upon binding to ECM components like fibronectin, collagen, or laminin, integrins undergo conformational changes, triggering “outside-in” signaling that promotes focal adhesion (FA) assembly, maturation and downstream intracellular signaling cascades [22]. A key signaling mediator in this process is the focal adhesion kinase (FAK), a cytoplasmic tyrosine kinase recruited to nascent FA sites through direct interaction with the integrin-associated protein paxillin [23]. Once recruited, FAK autophosphorylates at tyrosine 397 (Y397), creating a high-affinity binding site for SRC family kinases (SFKs) [24]. This interaction drives additional phosphorylation events that propagate downstream signaling, contributing to YAP/TAZ regulation.

While in physiological conditions upstream pathways ensure precise tuning of YAP/TAZ activity, their dysregulation may contribute to pathologies such as cancer and fibrosis, making YAP/TAZ attractive therapeutic targets for disease intervention. Thus, understanding the complex regulation of YAP/TAZ may provide valuable insights and offer potential strategies for targeting aberrant YAP/TAZ signaling in human disease. Toward this goal, we undertook CRISPR/Cas9-mediated loss-of-function screens in order to assess the phenotypic relevance of recently discovered YAP/TAZ regulators [25]. We identified FERMT2, a component of the integrin signaling pathway, as a new canonical regulator of YAP/TAZ activity.

## Results

### Genetic loss of function screens to identify YAP/TAZ regulators critical for tumor cells’ fitness

**Fig. 1:**
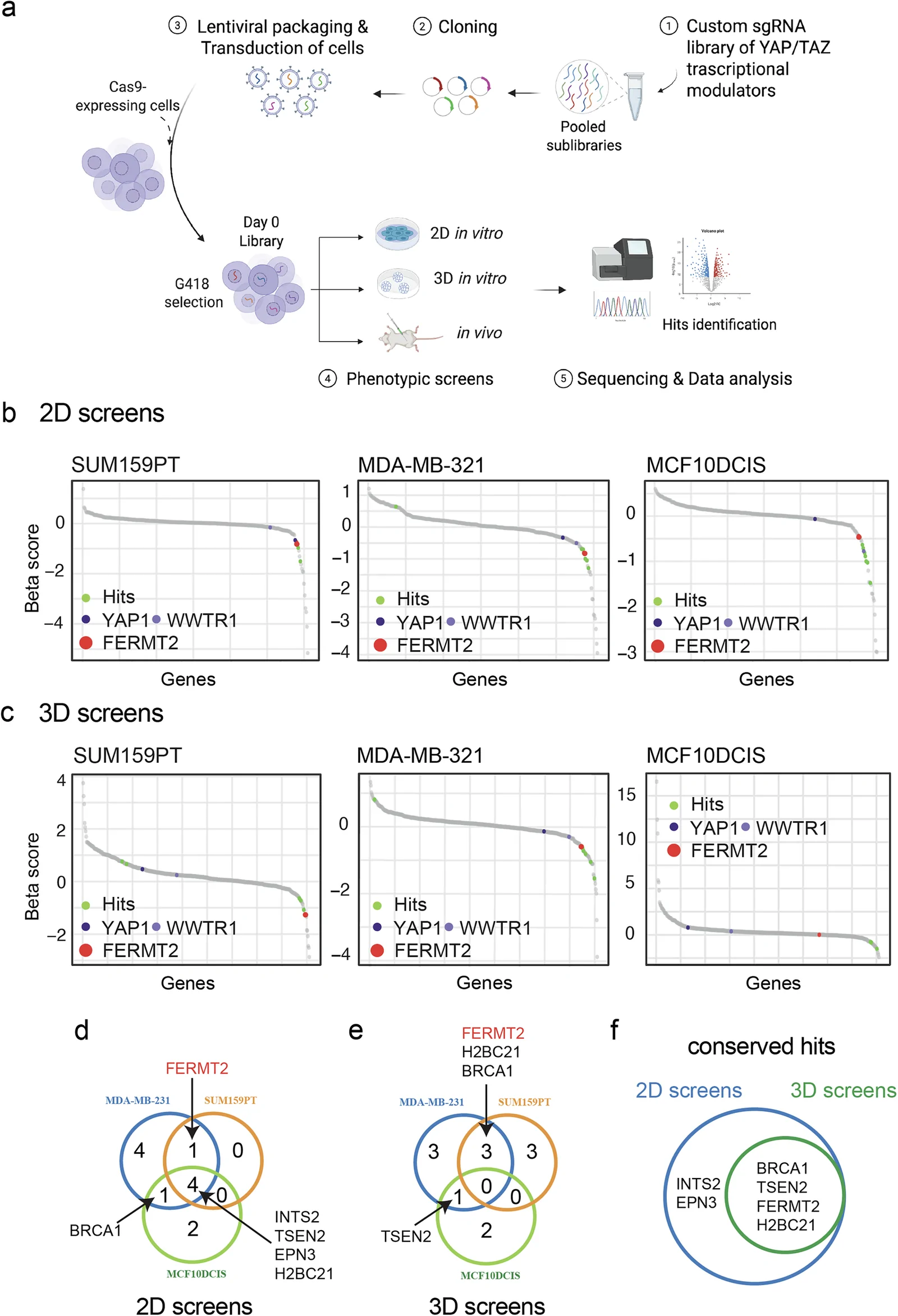
CRISPR/Cas9 loss of function screens. **a** Workflow of the sgRNA-based screens performed in adherent tissue culture conditions (2D), culturing cells in sphere-forming conditions (3D) or in vivo. Created with Biorender.com. **b** Waterfall plot of the gene-level MAGeCK analysis of the 2D screen, reporting the genes ranked by beta score (positive beta score = genes whose sgRNAs are enriched, negative beta score = genes whose sgRNAs are depleted). Green dots highlight genes with significant *P*-values (less than 0.05). FERMT2 is reported in red, YAP and TAZ in dark and light purple. **c** Waterfall plot of the gene-level MAGeCK analysis of the 3D screen, reporting the genes ranked by beta score (positive beta score = genes whose sgRNAs are enriched, negative beta score = genes whose sgRNAs are depleted). Greens dots highlight genes with significant *P* values (less than 0.05). FERMT2 is reported in red, YAP and TAZ in dark and light purple. Venn diagram of the genes identified as hits in the 2D screens (**d**) and 3D screens (**e**). **f** Venn diagram of the conserved hits (scoring in at least two out of the three cell lines) in 2D and 3D screens.

To uncover novel YAP/TAZ regulators relevant to breast cancer cell fitness, we leveraged our previous work, which identified genes critical for modulating their activity [25]. Based on these findings, we conducted CRISPR/Cas9 loss-of-function genetic screens to determine which of these YAP/TAZ regulators would be required for breast cancer cell fitness (Fig. 1a). We designed a viral sgRNA library targeting these 216 potential regulators, including 95 activators (genes whose loss reduced YAP/TAZ activity), 28 essential activators (genes whose loss reduced YAP/TAZ activity and cell viability), and 93 inhibitors [25]. The library was divided into sub-pools to enable flexible screen design and down-scaling required for in vivo conditions or clonal growth in suspension cultures. We also included sgRNAs targeting essential genes, which were used to assess Cas9-based genomic editing efficiency and establish viability thresholds (Fig. S1, Supplementary Data 1). Moreover, sgRNAs for known YAP/TAZ regulators served as an internal reference for hit calling, while non-targeting sgRNAs were used for passive barcoding, to monitor sgRNA representation drift unrelated to genome editing (Fig. S1 and Supplementary Data 1). We engineered three breast cancer cell lines to express Cas9: MDA-MB-231 and SUM159PT, representing aggressive triple-negative breast cancers (TNBC), and MCF10DCIS, an immortalized breast cancer line transformed by RAS overexpression, modeling ductal carcinomas [26].

First, we conducted a drop-out/drop-in screen in traditional two-dimensional tissue culture conditions (2D). Cells were transduced at a low MOI (T0) and subjected to antibiotic selection. After selection (PS), cells were cultured for up to seven passages (21 days). sgRNA representation was determined by sequencing. Significant sgRNA changes targeting a gene were identified using the MAGeCK algorithm [27] with a gene-level call for high stringency. Overall, we identified 12 genes, primarily drop-outs (i.e., genes promoting cell fitness) (Fig. 1b, d, Supplementary Data 1). Four genes (*INTS2*, *TSEN2*, *EPN3*, and *H2BC21*) were hit in all three cell lines, and two genes (*FERMT2* and *BRCA1*) were hit in two of the three cell lines (Fig. 1d).

Given YAP/TAZ role in anchorage-independent growth and survival, we also performed a screen in cells grown as spheroids in suspension cultures (3D). Due to limited sphere-forming efficiency (4-4.7% for MDA-MB-231 and MCF10DCIS; 7.5% for SUM159) (data not shown), the library was divided into three sub-pools, where sgRNA were sorted according to the previously identified categories of YAP/TAZ regulators (i.e., pool 1: activators, pool 2: inhibitors and pool 3: essential activators) [25]. We identified 12 fitness-related genes, four of which (*FERMT2*, *H2BC21*, *TSEN2*, and *BRCA1*) were hit in two of the three cell lines (Fig. 1c, e, Supplementary Data 1). Notably, there was a good degree of overlap among shared top 2D (six genes) and 3D hits (four genes), with four genes (*FERMT2*, *BRCA1*, *TSEN2*, and *H2BC21*) identified in both screens (Fig. 1f). Among these, *BRCA1*, *TSEN2*, and *H2BC21* are likely essential genes, as suggested by the unimodal distribution of their DepMap gene effect scores centered on negative values [28]. On the other hand, *FERMT2* is reported as a selective lethal gene, as indicated by the bimodal distribution of its gene effect scores.

**Fig. 2:**
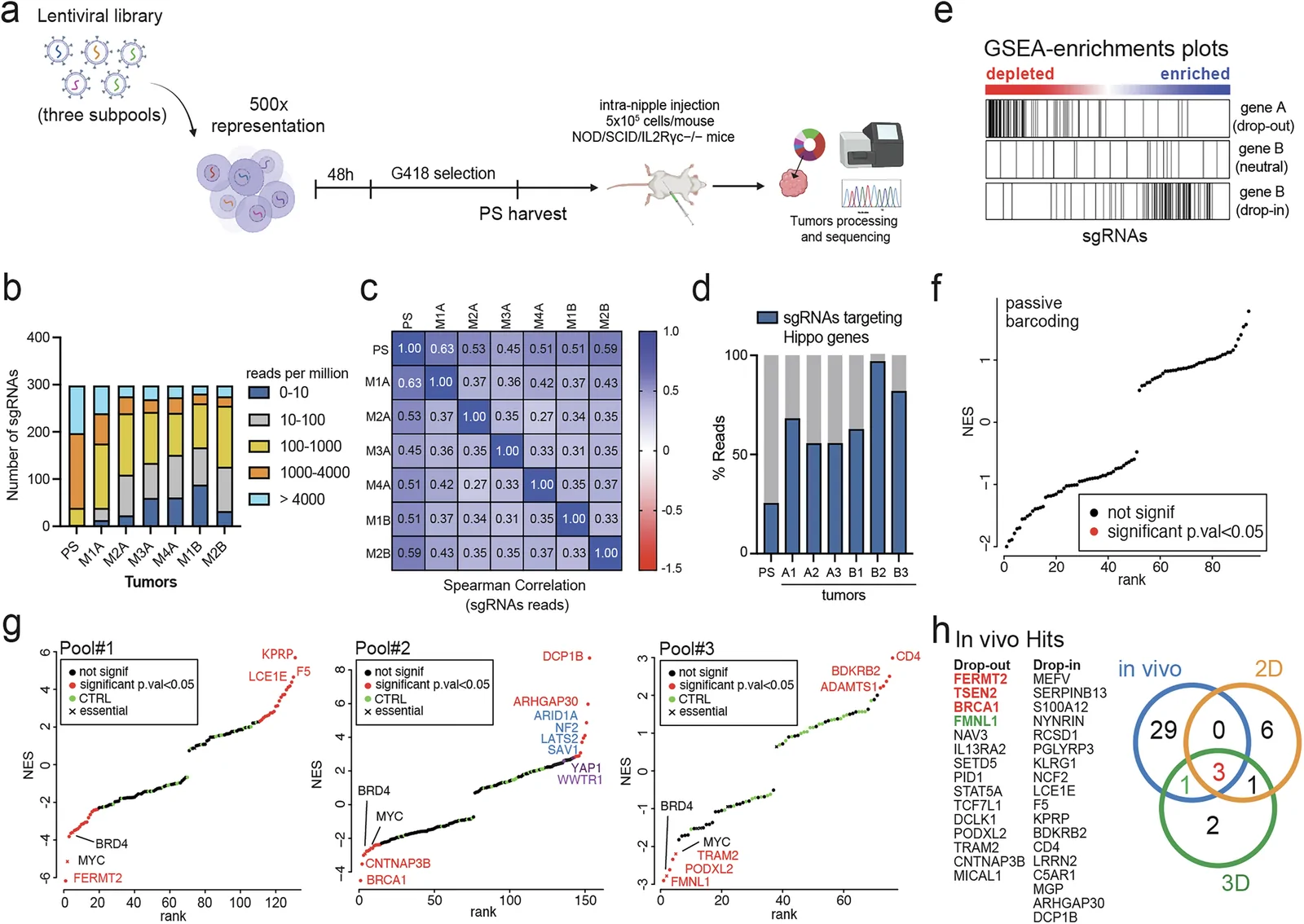
In vivo CRISPR-Cas9 loss of function screen. **a** Design of the in vivo sgRNA screen. **b** Passive barcoding experiment: bar plot of the distribution of sgRNAs in in vitro propagated transduced cells (PS=post-selection) and in different tumors (M1A-M2B) derived from the same cells xenografted in the mammary gland. Data shows that in vivo seeding shifts the sgRNA distribution (increases the fraction of sgRNAs with low representation) in the absence of Cas9 activity, yet the majority of the sgRNAs are still detected in cells derived from xenografts. **c** Spearman correlation matrix of the sgRNAs detected in the samples shown in (**b**). Despite the seeding bias altering sgRNA relative representations in xenografted cells (see **a**), the overall ranking is conserved, hence supporting the use of ranking-based algorithm to deconvolute in vivo sgRNA screens. **d** Stacked bar plot of the distribution of sgRNAs in cells prior to the in vivo injection (PS=post-selection) and in different xenografts. sgRNAs targeting Hippo genes are reported in blue. Loss of Hippo genes by Cas9 mediated editing provides a selective advantage in xenografts that leads to the enrichments of Hippo targeting sgRNAs. **e** Conceptual representation of how a GSEA-based ranking analysis can detect drop-out or drop-in sgRNA in a pooled sgRNA screen. The enrichment plots report the position (ranking) of each sgRNA (normalized reads) as vertical bars. sgRNA can cluster to the left (low abundance/depletion, hence negative Normalized Enrichment Score), to the right (high abundance/enrichment, hence positive Normalized Enrichment Score) or be evenly distributed (neither enriched nor depleted). The data is a cumulative report of all the tumors analyzed. **f** Waterfall plot of genes based on the cumulative ranking of their sgRNAs detected in xenografts from the passive barcoding experiments. Note that while genes could be ranked, no significant enrichment was detected by the GSEA-based ranking analysis. **g** Waterfall plot of the three sub-pooled screens. Red dots indicate significant hits. Green dots indicate non-targeting sgRNAs. MYC and BRD4 are two of the essential genes used as positive controls for efficient Cas9 editing and selective counter-selection of their sgRNAs due to competition. **h** On the left, list of the statistically significant drop-in and drop-out genes identified in the in vivo screen. On the right, Venn diagram of the overlap of the in vivo and the in vitro hits detected in MDA-MB-231 cells. In red are reported the hits consistently detected in the three screens, while in green those in common between the in vivo and the in vitro screens.

Next, we performed an in vivo screen in MDA-MB-231 cells: this cell line was chosen due to its high in vivo seeding efficiency (10%) and reliability for intra-nipple injection xenotransplants (Fig. 2a). Considering the in vivo screen challenges (seeding biases and random clonal drifts), we first assessed the feasibility of the design by examining whether in vivo seeding affected sgRNAs’ representation and distribution in tumors. To this end, we adopted a passive barcoding strategy: tumor cells not expressing Cas9 were transduced with a sub-pool of sgRNAs to compare sgRNA representation in tumors and in cells harvested post-selection, right before the injection. In this setting, fluctuations in sgRNAs are not expected, unless due to a seeding effect leading to subclonal growth advantage (and thus not linked to Cas9-mediated editing). Of note, sgRNA recovery in tumors was high (95% of sgRNAs with >10 reads/million), indicating that in vivo seeding and growth at the selected experimental conditions did not markedly bias the sgRNA library (Fig. 2b, Supplementary Data 2). Also, despite some sgRNA representation variability when comparing independent tumors, the overall sgRNA Spearman correlation across tumors was moderately high, indicating that a ranking-based metric could be used to deconvolute the in vivo screen (Fig. 2c). A further test in MDA-MB-231-Cas9 cells with sgRNA pools targeting YAP/TAZ regulators showed their overall enrichment (compared to pre-in vivo seeding), validating the system for detecting selective pressure altering sgRNAs’ enrichment (Fig. 2d). We applied Gene Set Enrichment Analysis (GSEA) to assess sgRNA depletion or enrichment, deriving the NES (normalized enrichment score) and significance for each gene (Fig. 2e). As expected, the passive barcoding experiment showed no significant sgRNA alterations in tumors compared to cells collected prior to the injection/PS (Fig. 2f).

The in vivo screen was performed by transducing MDA-MB-231-Cas9 cells with sgRNA sub-pools (three pools of 300-850 sgRNAs, Supplementary Data 2), similar to the 3D screen. Transduced and selected cells were injected into 6 mice (for the 300 sgRNA library) or 15 mice (for the 850 sgRNA library). GSEA identified sgRNAs targeting the essential genes *MYC* and *BRD4* as significantly depleted in tumors, while sgRNAs against Hippo genes (which are tumor suppressive) were significantly enriched, highlighting their importance for cancer cell survival (Fig. 2g and Fig. S2). Overall, we identified 33 hits (15 drop-outs and 18 drop-ins) (Fig. 2h, Supplementary Data 2). Three (*FERMT2*, *TSEN2*, and *BRCA1*) were also identified in the in vitro screens with the same cell line (Fig. 2h). Notably, sgRNAs targeting *FERMT2* were strongly depleted (Fig. 2g, h and Fig. S2).

### In-silico genetic analyses indicate that FERMT2 is a canonical regulator of YAP/TAZ and a selective liability

**Fig. 3:**
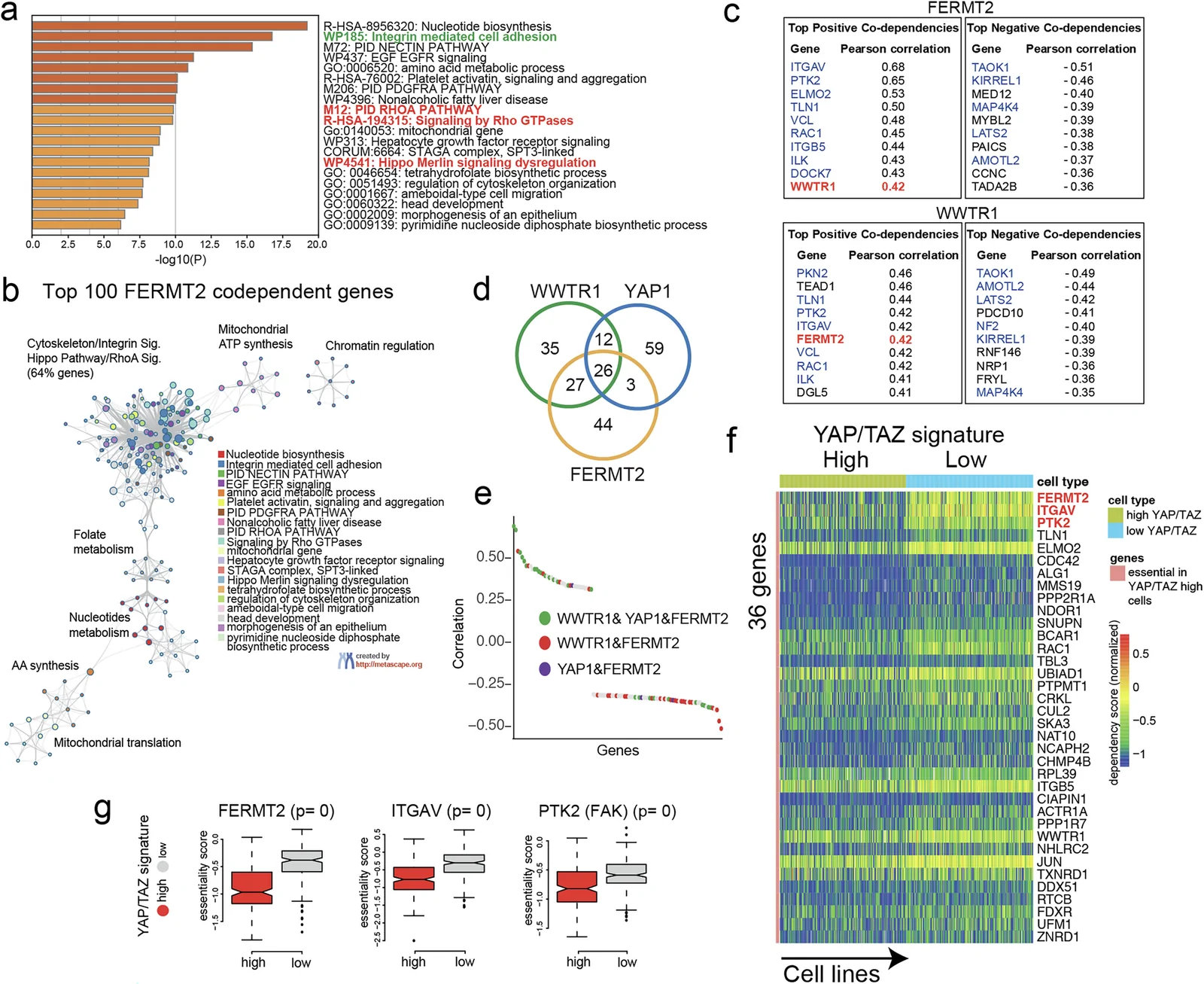
Bioinformatic analyses identify FERMT2 as a core regulator and genetic liability in YAP/TAZ-high cells. **a**, **b** GO analysis of the most enriched pathways in FERMT2 co-dependent genes. 96 genes out of the 100 were mapped to GO terms. **c** Top 10 positively and negatively correlated codependent genes for FERMT2 and TAZ (WWTR1). **d** Venn diagram of the shared codependent genes for FERMT2, WWTR1, and YAP. **e** Waterfall plot of FERMT2 codependent genes, color-coded when also codependent with YAP and TAZ (WWTR1). **f** Ranked heatmap of the normalized dependency score (essentiality) of the top 36 genes identified in silico as selectively essential in cells with high YAP/TAZ activity (YAP/TAZ high cells). A negative dependency score indicates that the gene is essential for a given cell line. **g** Box plot of essentiality score values for the three top-essential genes in YAP/TAZ high cells.

Given the consistent identification of FERMT2 as a top drop-out hit in our screens, we queried public datasets for potential genetic and functional links with YAP and TAZ. Analysis of breast cancer cohorts from METABRIC and TCGA via cBioPortal revealed a positive correlation between FERMT2 and both YAP1 and TAZ expression [29, 30] (Fig. S3). Next, we mined the DepMap database [28] to assess whether FERMT2 was genetically linked to TAZ or YAP. Here, we leveraged co-dependency analysis, which exploits the correlation of the essentiality profiles of genes pairs across a large panel of cancer cell lines to infer their functional cooperation. Analysis of the top 100 FERMT2 co-dependent genes revealed a strong enrichment for genes involved in integrin signaling, cytoskeletal regulation, and the Hippo pathway (Fig. 3a, b; Fig. S4; Supplementary Data 3). A large fraction (56%) of FERMT2 co-dependent genes overlapped with the top co-dependent genes of YAP or TAZ (WWTR1), including FERMT2 *(*co-dependent in TAZ top 100) and TAZ/WWTR1 (top FERMT2 co-dependent) (Fig. 3c, d and Fig. S5). These shared FERMT2/YAP/WWTR1 co-dependent genes were among the top correlated co-dependencies for FERMT2 (Fig. 3e), thus indicating strong genetic relationships among the three genes.

We also interrogated the DepMap database to unveil genetic dependencies in cells with high YAP/TAZ activity (i.e., genes that are selectively required to support the fitness of cells with high YAP/TAZ activity) [28]. Using a YAP/TAZ expression signature [10, 31] (preemptively purged of proliferation-associated genes) as a proxy, we categorized cell lines as exhibiting high (YAP/TAZ signature high) or low (YAP/TAZ signature low) expression of YAP/TAZ-regulated genes (Fig. S6a and Supplementary Data 3).

We then identified genes with selective co-dependencies in YAP/TAZ-high cells as those required for survival specifically in these cells, but not (or less so) in YAP/TAZ-low cells.

This analysis identified 36 co-dependent genes in YAP/TAZ high cells (Fig. 3f, Fig. S6b and Supplementary Data 3). GO analysis revealed strong enrichment for genes involved in cell adhesion and integrin signaling (Fig. S7), with *FERMT2* as the top co-dependent gene, along with *ITGAV* (integrin receptor subunit) and *PTK2* (FAK) (Fig. 3f, g and Supplementary Data 3). Overall, the in silico genetic analyses corroborated our screen results, indicating that FERMT2 is a core component of signaling pathways regulating YAP/TAZ and is required for the fitness of cells with elevated YAP/TAZ activity.

### Experimental deconvolution confirms that FERMT2 is required for in vitro and in vivo fitness of triple-negative breast cancer cells

**Fig. 4:**
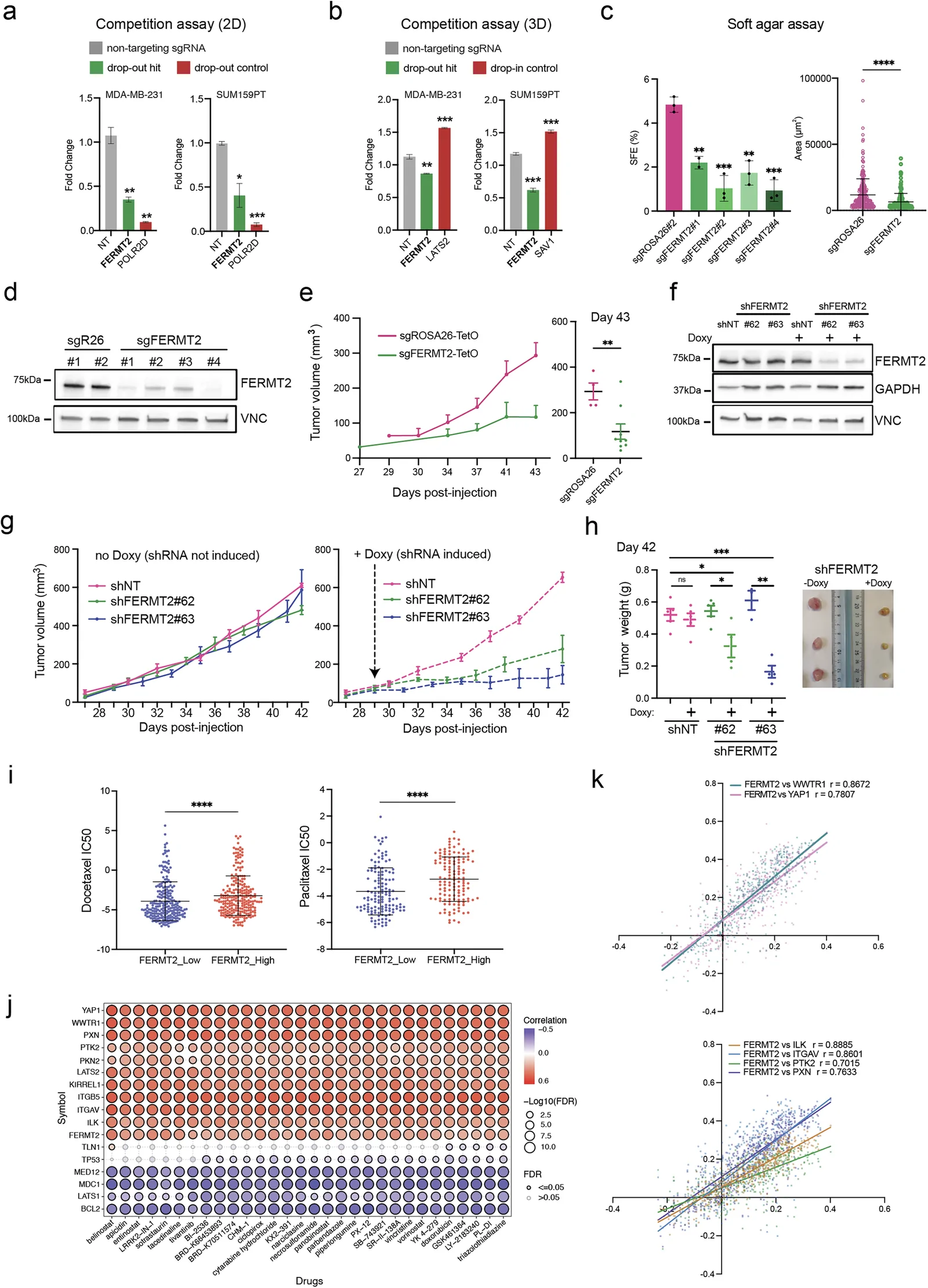
Loss of FERMT2 compromises tumor cell growth. **a** Competition assay of MDA-MB-231 (left panel) or SUM159PT cells (right panel) grown in adhesion (2D). In red, drop-out control targeting the essential POLR2D gene. Data are represented as mean ± SD, *n* = 2. *p*-values are calculated by two-tailed t-test between the non-targeting sgRNAs (sgNT) and gene-targeting sgRNAs. **b** Competition assay of cells grown in suspension (3D) for four passages. In red, drop-in controls targeting the tumor suppressor genes LATS2 and SAV1. Data are represented as mean ± SD, *n* = 3. Statistical significance was calculated on P4 (two-tailed t-test). **c** Soft agar assay of SUM159PT-Cas9 cells transduced with the indicated sgRNAs. The sphere forming efficiency (SFE) was assessed after 10 days of culture. Right panel: scatter dot plot of the colony area, *n* = 3. Two-tailed t-test was applied to determine the statistical significance. **d** Western Blotting analysis of MDA-MB-231-Cas9 cells transduced with the indicated sgRNAs. VNC was used as a loading control. **e** Tumor growth of MDA-MB-231-Cas9 cells transduced with the indicated sgRNAs. Cells were xenografted in the mammary gland of NSG mice (*n* = 4 sgROSA#26, *n* = 9 sgFERMT2#1 or #4). **f** Western Blotting analysis of MDA-MB-231 cells transduced with tet-regulated shRNAs. Doxycycline (Doxy) was used to induce the expression of the indicated shRNAs. shNT: non-targeting shRNA. VNC was used as a loading control. **g** Tumor growth of MDA-MB-231 cells transduced with the indicated shRNAs. Cells were xenografted in the mammary gland of NSG mice. Left panel: tumor growth in not-induced conditions (no-doxycycline). Right panel: tumor growth upon doxycycline administration (*n* = 4/5 mice for each experimental group). The arrow indicates the time when doxycycline administration started. **h** Left panel: weight of the tumors collected at the endpoint. Two-tailed t-test was applied to determine the statistical significance. Right panel: representative pictures of dissected tumors. **i** Scatter dot plot of Docetaxel (lef) and Paclitaxel (right) IC50 values for cancer cell lines with high (FERMT2_high) or low (FERMT2_low) expression of FERMT2. Statistical significance was assessed by two-tailed t-test. **j** Heatmap of the correlation values between IC50 and expression of the indicated genes (Y axis) and drugs (x axis). Red indicates a positive correlation (higher expression associated with increased drug resistance), while blue indicates negative correlation (higher expression associated with increased drug sensitivity). **k** Spearman correlation between gene-drug sensitivity correlation values for FERMT2 (x axis) and select genes (y axis).

The consistency observed across our screening and bioinformatic analyses, along with previous studies implicating FERMT2 in TNBC [32, 33] and in other tumor types [32, 34–38], prompted further investigation. To validate the FERMT2-KO phenotype, we first conducted competition assays in MDA-MB-231 and SUM159PT cell lines. Cas9-expressing cells were transduced with the pool of sgRNAs targeting FERMT2 (sgFERMT2) used in the screens, with non-targeting sgRNAs (sgROSA26, targeting the murine ROSA26 locus), sgRNAs targeting the essential RNA Pol II subunit (sgPOLR2D) as drop-out control, or sgRNAs targeting established YAP/TAZ inhibitors (sgLATS1, sgSAV1) as drop-in controls. In 2D cultures, sgFERMT2-bearing cells were outcompeted in both cell lines (Fig. 4a). Similarly, competitive growth assays in spheroid cultures showed out-competition of sgFERMT2-expressing cells (Fig. 4b). Loss of FERMT2 also impaired tumorigenic growth in soft-agar assays, resulting in reduced colony-forming efficiency and size (Fig. 4c, d), confirming previous reports [32, 39]. In addition, orthotopic xenografts of FERMT2-KO MDA-MB-231 cells showed strong impairment of tumor growth (Fig. 4e), as previously observed in distinct in vivo models of breast cancer [32, 39–41], melanoma [42, 43] and lung adenocarcinoma [37]. We then asked whether acute loss of FERMT2 would hamper tumor growth. To this end, we transduced MDA-MB-231 cells with two doxycycline-regulated shRNAs targeting FERMT2 or a non-targeting shRNA (shNT) (Fig. 4f). Upon detection of the initial growth of the tumor mass, half of the xenografted mice were fed with doxycycline containing food, in order to induce the expression of the shRNAs. As expected, in mice fed with standard food (i.e., no shRNA induction), both shNT and shFERMT2 tumors grew comparably (Fig. 4g, left panel). On the other hand, in the doxycycline-treated cohorts, growth of shFERMT2 tumors was blunted (Fig. 4g, right panel, and Fig. 4h), indicating a strong requirement for FERMT2 expression. Overall, our data emphasizes the role of FERMT2 in supporting growth and fitness of breast cancer cells.

To further establish the clinical relevance of FERMT2 in cancer, we analyzed publicly available datasets from TCGA. Notably, missense mutations in FERMT2 were associated with improved patient prognosis, suggesting that loss of FERMT2 function may attenuate tumor aggressiveness (Fig. S8a). Moreover, given that the activation of YAP/TAZ or FAK has been linked to chemoresistance [5, 44], we explored whether FERMT2 could modulate responses to chemotherapeutic drugs such as taxanes, which are frontline chemotherapeutics in TNBCs. Indeed, loss of FERMT2 sensitized MDA-MB-231 cells to taxol (Fig. S8b). In addition, while naive SUM159PT were highly sensitive to taxol and therefore not further sensitized by FERMT2 loss (Fig. S8c), long-term resistant SUM159PT (SUM159PT^LTR^), a taxol chemoresistant derivative [45], exhibited chemosensitization upon FERMT2 loss (Fig. S8d). These observations were further supported by pan-cancer analysis showing that cancer cell lines with low FERMT2 expression are more sensitive to taxanes than those expressing high level of FERMT2 (Fig. 4i). Consistent with these data, a recent publication reported increased taxol resistance by tumors expressing elevated levels of FERMT2 [46]. Building on this evidence, we next asked whether FERMT2 may modulate chemosensitization/chemoresistance in ways similar to either YAP/TAZ activation or integrin-dependent FAK activation. To this end, we exploited the Gene Set Cancer Analysis (GSCA) platform [47], which leverages the Cancer Therapeutics Response Portal (CTRP) dataset, to extract correlations between the expression of a selected gene and the IC50 values for a large panel of drugs across an extensive collection of tumor cell lines [48]. By comparing correlation profiles for all drugs in the database, we assessed whether altered expression of Integrin signaling genes (including FAK and FERMT2) and YAP/TAZ might be associated with similar drug response patterns, to infer similarities in chemoresistance modulation and mechanism of action. Remarkably, FERMT2 drugs correlation profiles showed strong concordance with those of YAP/TAZ and of integrin signaling components (Fig. 4j, k). This shared pattern of drug sensitivity would be coherent with a concordant role of the FERMT2-FAK-YAP/TAZ pathway in modulating chemoresistance.

Altogether, these data support that FERMT2 is a cancer fitness gene and suggest it may modulate tumor aggressiveness and drug responses.

### FERMT2 activates YAP/TAZ independently of the Hippo pathway

**Fig. 5:**
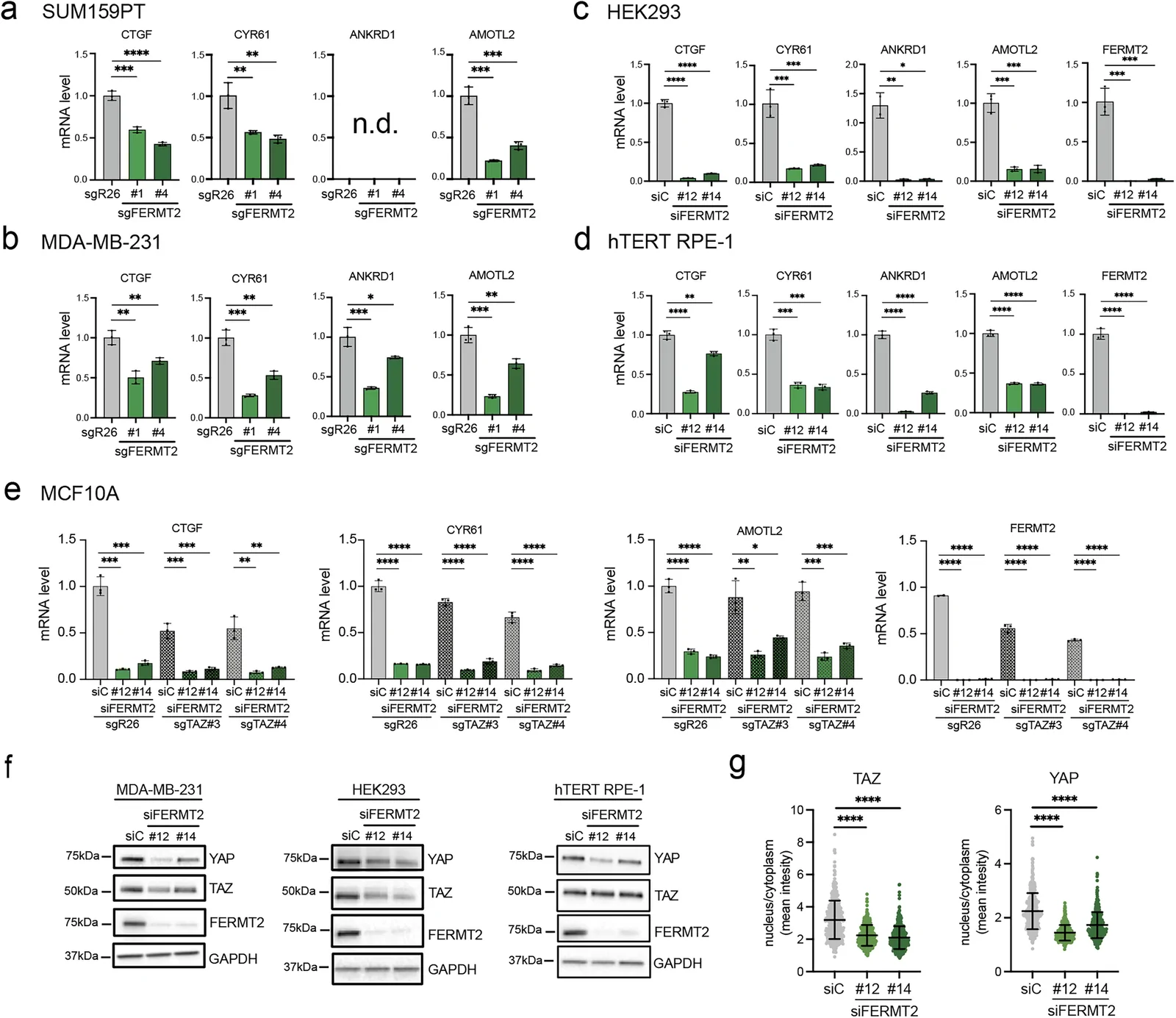
FERMT2 regulates YAP/TAZ activity. **a**–**d** *FERMT2* was knocked out or silenced, and the activity of YAP/TAZ was assessed by RT-qPCR analysis of canonical targets (*CTGF*, *CYR61*, *ANKRD1*, and *AMOTL2*). **a**, **b** Cas9-expressing breast cancer cell lines were transduced with sgRNAs targeting *FERMT2* or a sgRNA targeting the Rosa 26 locus (sgR26, non-targeting sgRNA). mRNA levels of YAP/TAZ targets were assessed by RT-qPCR. N.d.: not detected. **c**, **d** HEK293 and hTERT RPE-1 cells were transfected with the indicated siRNAs. siC=non-targeting siRNA (negative control). **e **Evaluation of YAP/TAZ targets in MCF10A-Cas9 cells mock edited (sgR26) or transduced with two sgRNAs targeting *TAZ* (sgTAZ#3, #4). **f** Evaluation of FERMT2, YAP, and TAZ levels by western-blotting analyses of cells transfected with siFERMT2. GAPDH was used as loading control. **g** Nuclear-to-cytoplasmic mean intensity ratio in spinning disk confocal images for TAZ (left) and YAP (right) upon FERMT2 silencing in hTERT RPE-1 cells. Sample size 360-420 cells per condition. Two-tailed t-test was used throughout.

FERMT2 has been previously implicated in the regulation of YAP and TAZ, though evidence is conflicting as to whether FERMT2 modulates both proteins and the associated pathways [49–52]. Our previous screen showed that FERMT2 silencing downregulated the activity of ectopically expressed TAZ in a luciferase reporter assay [25], suggesting that FERMT2 may support YAP/TAZ activity (Fig. S9). To clarify FERMT2 role in YAP/TAZ regulation, we conducted loss-of-function experiments using either Cas9 editing or siRNA silencing. FERMT2-depleted cells displayed reduced expression of classical YAP/TAZ target genes (Fig. 5a, b). This evidence was confirmed by FERMT2 silencing in both breast cancer (Fig. S10a, b) and non-breast immortalized cell lines (Fig. 5c, d), indicating that this regulation is not breast cancer-specific, consistent with previous studies in mesenchymal stem cells [53], renal [53] and Sertoli cells [50]. Furthermore, silencing of FERMT2 in TAZ-KO cells still led to downregulation of YAP/TAZ targets (Fig. 5e and Fig. S10c), thus implying that FERMT2 regulates both YAP and TAZ.

YAP and TAZ activity is primarily controlled post-translationally via nuclear shuttling and protein accumulation. Consistent with decreased YAP/TAZ activity upon FERMT2 loss, a reduction in YAP and TAZ protein level was observed (Fig. 5f). Moreover, FERMT2 loss diminished YAP/TAZ nuclear accumulation (Fig. 5g), suggesting that altered protein turnover and localization contribute to the downregulation of YAP/TAZ activity.

**Fig. 6:**
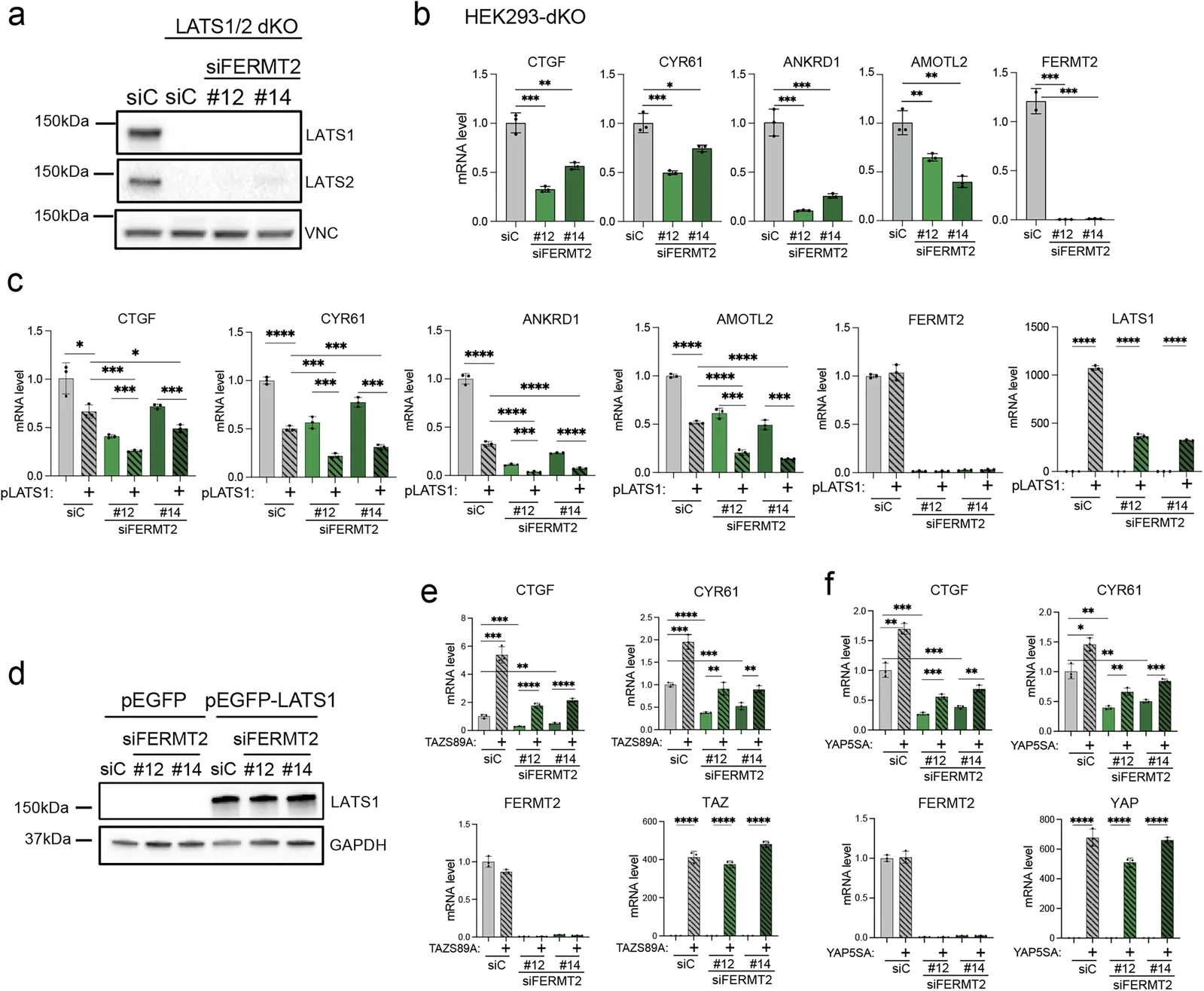
FERMT2 regulates YAP/TAZ independently of the Hippo pathway. **a** Western Blotting analysis of HEK293 cells knock-out for LATS1 and 2 (HEK293-dKO cells). VNC was used as a loading control. **b** RT-qPCR analysis of YAP/TAZ canonical targets in HEK293-dKO cells. Cells were transfected with siRNAs targeting *FERMT2* (siFERMT2#12 and #14) or mock silencing siRNA (siC). **c** RT-qPCR analysis of YAP/TAZ canonical targets in HEK293-dKO cells complemented by ectopic expression of LATS1 and transfected with the indicated siRNAs. **d** Western Blotting analysis of HEK293-dKO cells transfected with exogenous LATS1 and with siRNAs targeting FERMT2. GAPDH was used as the loading control. RT-qPCR analysis of YAP/TAZ targets in HEK293-dKO cells transfected with pcDNA3-HA-TAZ^S89A^ (**e**) or pQCXIH-Myc-YAP^5SA^ (**f**) and with siRNAs targeting *FERMT2* (siFERMT2#12 and #14) or mock silencing siRNA (siC). All data are represented as mean ± SD, *n* = 3. Two-tailed t-test was used throughout.

**Fig. 7:**
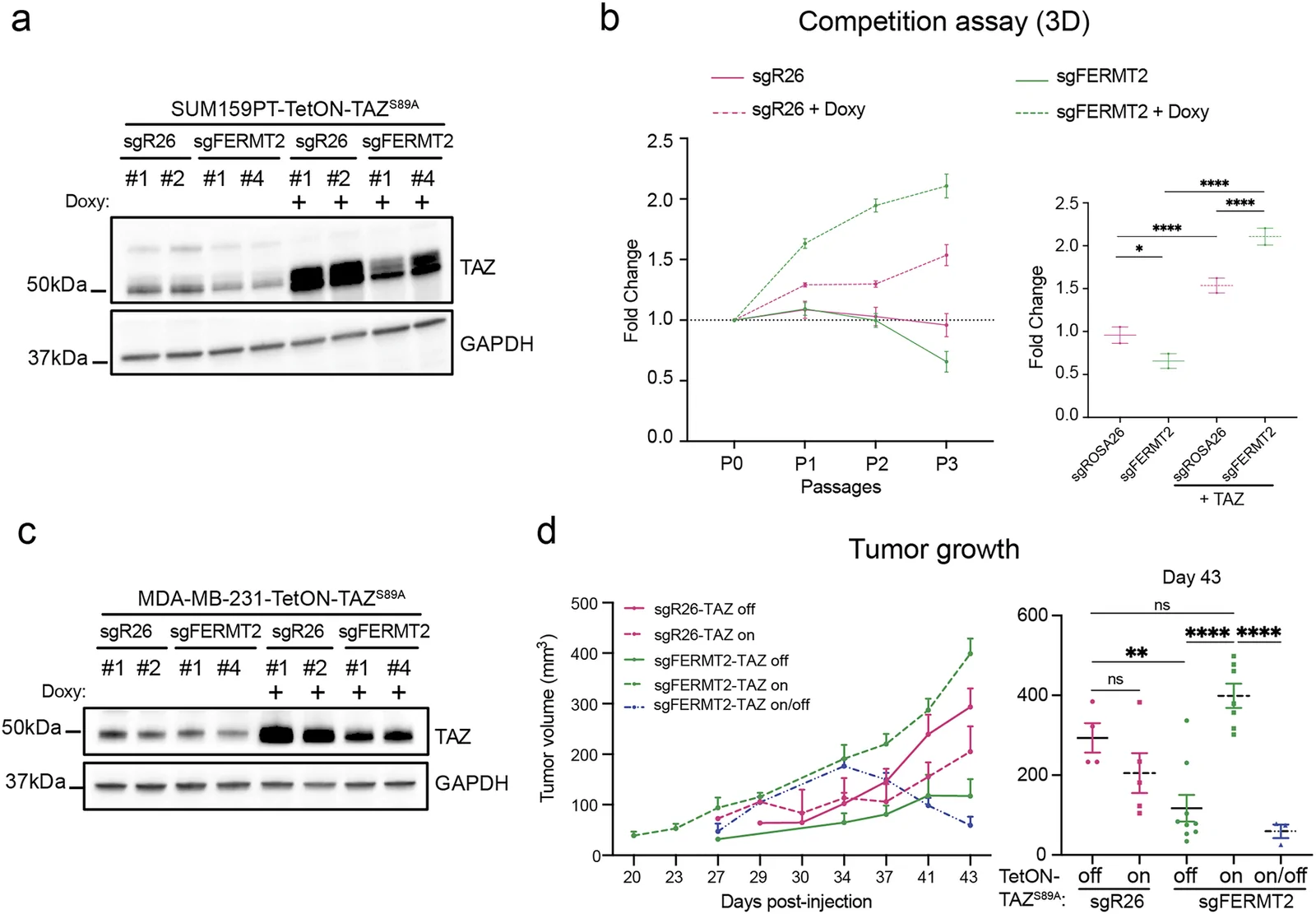
Ectopic expression of TAZ rescues growth of FERMT2-KO cells. **a** Western Blotting analysis of SUM159PT-rtTA-Cas9 transduced with tetON-TAZ^S89A^. GAPDH was used as a loading control. The expression of TAZ was induced by adding 500 ng/ml doxycycline (Doxy) to the tissue culture medium. **b** Competition assay of SUM159PT-rtTA-Cas9 cells grown in suspension (3D). *N* = 3 (independent cultures with two different sgRNAs targeting the same gene). Statistical analyses were performed by two-way ANOVA with Tukey’s multiple comparison test. **c** Western Blotting analysis of MDA-MB-231-Cas9 cells transduced with tetON-TAZ^S89A^. GAPDH was used as a loading control. The expression of TAZ was induced by adding 500 ng/ml doxycycline (Doxy) to the tissue culture medium. **d** Tumor growth of MDA-MB-231-Cas9 cells, mock edited or FERMT2-KO, transduced with tetON-TAZ^S89A^. Cells were xenografted in the mammary gland of NSG mice. Left panel: tumor growth in absence (solid line) or presence of TAZ^S89A^ induction (dashed line) in sgROSA26 (sgR26, pink) or sgFERMT2 (green) xenografts (*n* = 4/5 sgR26-TAZ on and sgR26-TAZ off, *n* = 7/9 sgFERMT2-TAZ on and sgFERMT2-TAZ off). In blue, drop of tumor growth upon doxycycline withdrawal on day 34 in sgFERMT2-TAZ on/off mice (*n* = 3). Right panel: tumor volume on day 43. Two-tailed t-test was applied to determine the statistical significance.

Considering that the Hippo pathway is a major YAP/TAZ regulator, we investigated whether FERMT2 may control YAP/TAZ through this pathway. To this end, we employed LATS1/2 double-KO (LATS-dKO) cells, where YAP/TAZ are active and freed from Hippo regulation (Fig. 6a). Notably, FERMT2 loss in LATS-dKO cells still hampered YAP/TAZ activity, as shown by reduced YAP/TAZ target gene expression (Fig. 6b). As expected, ectopic expression of LATS1 in LATS-dKO cells repressed YAP/TAZ target genes, confirming that the Hippo pathway was functional (Fig. 6c, d). This reduction was further pronounced by FERMT2 silencing (Fig. 6c), supporting the hypothesis that FERMT2 regulates YAP/TAZ independently of Hippo signaling. On the other hand, overexpression of YAP and TAZ rescued YAP/TAZ target expression in both control and FERMT2-depleted cells (Fig. 6e, f), confirming that YAP/TAZ are downstream effectors of FERMT2. Collectively, these data indicate that FERMT2 and the Hippo pathway independently regulate YAP/TAZ, with FERMT2 exerting an epistatic effect over Hippo. These findings also imply that FERMT2 is required to activate YAP/TAZ and support cell growth and, consequently, that its loss may impair cancer cell growth due to defective YAP/TAZ activity. To verify this, we engineered cells for doxycycline dependent expression of an activated TAZ mutant, TAZ^S89A^ [25]. Ectopic expression of TAZ^S89A^ rescued the growth of FERMT2-KO cells both in vitro and in vivo (Fig. 7a–d). Of note, this rescue depended on continuous TAZ expression, as FERMT2-KO tumors regressed upon doxycycline withdrawal at day 34 (Fig. 7d, blue line).

Overall, these data indicate that FERMT2 supports cancer cell fitness by sustaining YAP/TAZ activity. As a side note, ectopic expression of TAZ^S89A^ conferred FERMT2-KO cells a reproducible stronger phenotypic rescue both in vitro and in vivo (Fig. 7b, d). While counterintuitive at first, considering that TAZ^S89A^ levels, albeit higher than the endogenous, are reduced in FERMT2-KO compared to wild type cells, this may reflect a reduced intrinsic tumor suppression. Seemingly paradoxical observations have been reported for other oncogenes, that upon strong overexpression/activity elicit intrinsic antiproliferative/pro-apoptotic responses [54].

### FERMT2 promotes YAP/TAZ activation through FAK

**Fig. 8:**
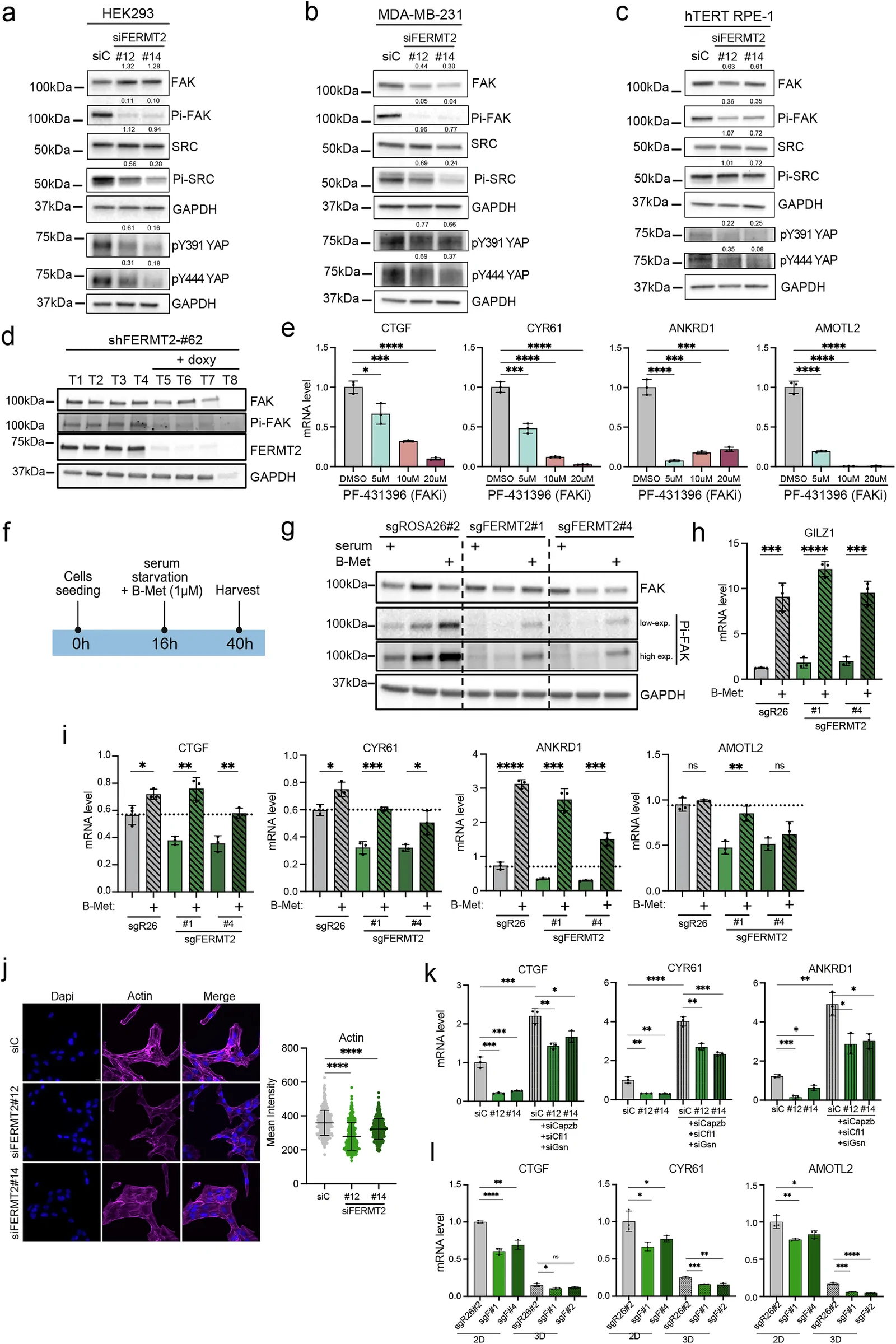
Activation of YAP/TAZ by FERMT2 is FAK-dependent. **a**–**c** Western Blotting analysis of cells transfected with the indicated siRNAs targeting FERMT2. GAPDH was used as a loading control. Normalized densitometric values are reported above relevant signals. **d** Western Blotting analysis of MDA-MB-231 shFERMT2 xenografts. GAPDH was used as the loading control. **e** RT-qPCR analysis of canonical YAP/TAZ targets performed in MDA-MB-231 cells treated with the FAK inhibitor PF-431396 at the indicated concentration for 24 hours. **f** Outline of the protocol used for inducing FAK activity using the glucocorticoid betamethasone (B-Met). **g** Western Blotting analysis of MDA-MB-231-Cas9 treated with betamethasone. GAPDH was used as the loading control. RT-qPCR analysis of a glucocorticoid responsive gene, *GILZ1* (**h**) and canonical YAP/TAZ targets (**i**). **j** Left panel, representative spinning disk confocal images of hTERT RPE-1 cells FERMT2 silenced (siFERMT2#12 and #14) or mock silenced (siC). Actin fibers were stained with phalloidin (F-actin signal shown in purple), while nuclei were stained with Dapi. Sample size 285-370 cells per condition. Scale bar 100 uM. Right panel, mean intensity of F-actin signals (phalloidin). **k** Evaluation of YAP/TAZ targets in MCF10A cells transfected with siRNAs targeting *FERMT2* (siFERMT2#12 and #14) or mock silencing siRNA (siC), alone or in combination with siRNAs targeting CapZ, Cofilin and Gelsolin. **l** Evaluation of YAP/TAZ targets in SUM159PT-rtTA-Cas9 mock edited (sgR26) or transduced with two sgRNAs targeting FERMT2 (sgF#1, sgF#4), grown either in adhesion (2D) or in suspension (3D). All data are represented as mean ± SD, *n* = 3. Two-tailed t-test was used throughout.

Our genetic analysis in LATS-dKO cells indicated that FERMT2 regulates YAP/TAZ independently of the Hippo pathway, thus suggesting an alternative regulatory mechanism. FERMT2 is an adaptor protein that associates with integrin receptors to promote paxillin recruitment and FAK activation [55]. Given the role of integrin-mediated focal adhesion signaling in regulating YAP/TAZ across various cancer contexts [16, 56], we investigated whether FERMT2 loss impaired FAK activation and signaling. FERMT2 silencing resulted in loss of FAK phosphorylation (Tyr397) and reduced SRC activation (Tyr416) in several cell lines, indicating that FERMT2 is required to support FAK signaling (Fig. 8a–c). This observation was further confirmed in tumors upon FERMT2 silencing (Fig. 8d).

Both YAP and TAZ are reported to be phosphorylated on tyrosine residues and activated by FAK and its downstream effector SRC [53, 56–59]. To assess whether FERMT2 loss would reduce YAP phosphorylation, we took advantage of available antibodies against mapped tyrosine phosphorylation sites on YAP [57]. Silencing of FERMT2 decreased YAP phosphorylation on both Y391 and Y444 residues (Fig. 8a–c), indicating that FAK is a downstream effector of FERMT2 necessary for YAP/TAZ activation. Accordingly, pharmacological inhibition of FAK with two small molecule inhibitors, PND-1186 and PF-431396 (Fig. S11a), impaired YAP/TAZ target genes expression (Fig. 8e and Fig. S11a, b), further supporting that FAK activity is required to sustain YAP/TAZ target transcription. Since glucocorticoids can activate FAK in breast cancer cells [17], we hypothesized that, if FAK acts downstream of FERMT2, glucocorticoid-mediated FAK activation may rescue YAP/TAZ activity in FERMT2-depleted cells. We thus treated serum-starved mock-control (sgROSA26) and FERMT2-KO cells with the glucocorticoid betamethasone, as previously described [17] (Fig. 8f). Serum starvation blunted FAK activation, as assessed by its phosphorylation (Fig. 8g). Importantly, betamethasone treatment activated FAK in both mock-control and FERMT2-KO cells (Fig. 8g). Of note, glucocorticoid signaling was functional in FERMT2-KO cells, as indicated by the activation of GILZ1, a glucocorticoid responsive gene (Fig. 8h). As reported, betamethasone induced YAP/TAZ target genes expression in mock-control cells, but, more importantly, rescued their transcription also in FERMT2-KO cells, indicating that FAK activation, downstream of FERMT2, is key in YAP/TAZ regulation (Fig. 8i).

### Loss of FERMT2 is partially epistatic over actin-driven mechanosignaling

Considering that FERMT2, together with Paxillin and Talin, links integrin receptors to the actin cytoskeleton, we next assessed whether loss of FERMT2 (and consequent bypass of FAK activation) could hinder YAP/TAZ activity by impairing mechanosignaling. Notably, transient or stable depletion of FERMT2 in all the tested cell lines did not markedly affect cell morphology, size, or adhesion, as assessed during routine culturing of the FERMT2-KO cell lines. Indeed, evaluation of actin filaments in FERMT2-silenced cells revealed only a mild decrease in global actin polymerization, suggesting that FERMT2 plays a limited role in maintaining or establishing actin filaments (F-actin) (Fig. 8j). To further explore potential epistatic relations of actin polymerization and FERMT2 in regulating YAP/TAZ, we genetically enhanced F-actin formation by silencing the actin-capping and -severing proteins CapZ, Cofilin, and Gelsolin (Fig. S12) [9]. As reported [9], silencing these F-actin inhibitors activated YAP/TAZ (Fig. 8k). Combined silencing of FERMT2 and F-actin inhibitors resulted in decreased YAP/TAZ target gene expression; however, this reduction was less pronounced than that observed upon FERMT2 depletion alone, thus indicating a partial epistasis of FERMT2 over F-actin polymerization (Fig. 8k).

In contrast, culturing cells in suspension—thereby relieving mechanical tension—strongly suppressed YAP/TAZ activity (Fig. 8l). Under these conditions, FERMT2 depletion caused a further, albeit mild, decrease in YAP/TAZ expression (Fig. 8l). While statistically significant, this reduction was modest compared to the dramatic inhibition of YAP/TAZ activity induced by loss of adhesion.

Overall, these data suggest that FERMT2 can partially act through F-actin–mediated mechanotransduction to modulate YAP/TAZ signaling. This could indicate that FERMT2 may influence mechanotransduction not solely via partial modulation of actin dynamics but potentially through additional mechanosensitive pathways. Further investigation will be required to fully elucidate how FERMT2-driven signaling interfaces with actin and other mechanotransducers.

## Discussion

Here we have provided compelling evidence that FERMT2 is a critical activator of YAP and TAZ, functioning through integrin–FAK signaling. FERMT2 (Fermitin Family Member 2, also known as Kindlin-2) is an adaptor protein that plays a central role in integrin-mediated signaling and focal adhesion assembly [60–63]. It activates FAK by interacting with integrins and other focal adhesion proteins to stabilize signaling complexes at adhesion sites. This promotes integrin clustering and tonic FAK activation (by autophosphorylation at Y397), thus facilitating SRC recruitment and amplifying downstream signaling that controls cytoskeletal dynamics, motility, and survival. By coordinating integrin-FAK signaling, FERMT2 contributes to cell migration, invasion, and response to substrate rigidity, enabling cells to sense and adapt to mechanical cues from the extracellular matrix [18, 55]. In addition, FERMT2 supports integrin turnover and recycling, conferring adhesion plasticity essential for tissue repair and homeostasis [60–63]. Consequently, FERMT2 dysregulation has been linked to cancer progression, fibrosis, and other pathologies involving altered adhesion and motility [64].

Previous studies have implicated FERMT2 in the regulation of either YAP or TAZ, but with inconsistent findings regarding its target (YAP vs TAZ), pathway involvement (Hippo-dependent or -independent), and leaving unaddressed whether FERMT2 serves as a general regulator of YAP/TAZ or rather it functions in a tissue- or context-specific manner [49–52]. Here, we reconcile these observations by providing experimental evidence and in silico analyses showing that FERMT2 acts as an upstream activator of both YAP and TAZ across diverse contexts. Our data indicate that the loss of FERMT2 leads to reduced engagement of FAK, which in turn is required for the activation of YAP and TAZ, independently of the Hippo pathway. Notably, cells with high YAP/TAZ activity display increased reliance on FERMT2, integrin signaling, and FAK, suggesting selective vulnerabilities that may be therapeutically actionable. These findings align with recent studies highlighting the role of FAK signaling in supporting YAP activity in TNBC and other tumor types [17, 53, 56, 65–67].

Crucially, the impaired fitness and eventual demise of FERMT2-deficient cells can be attributed specifically to the loss of YAP and TAZ activity, as evidenced by the ability of an activated TAZ variant to fully rescue cell growth in the absence of FERMT2. This suggests that FERMT2 loss primarily compromises cell fitness by disrupting YAP/TAZ activation rather than through other potential downstream effectors of the integrin-FAK signaling. These findings point to the essential role of FERMT2 activity and suggest that the loss of YAP and TAZ signaling is the primary cause of tumor cells’ demise due to FERMT2 loss. Overall, the strong genetic dependencies between FERMT2, YAP, and TAZ offer potential therapeutic opportunities: targeting FERMT2 and its downstream effectors could be leveraged for synthetic lethality-based treatments aimed at neutralizing YAP/TAZ hyperactivity, which is particularly relevant for poorly differentiated and aggressive tumors that often exhibit hallmarks of YAP/TAZ overactivation. In these contexts, it might be worthwhile to assess the efficacy of the FAK inhibitors that are currently under evaluation in both preclinical and clinical settings [68, 69].

The regulation of YAP/TAZ by FERMT2 occurs independently of the canonical Hippo pathway. Indeed, the relief of Hippo-mediated repression in FERMT2-depleted cells is insufficient to activate YAP/TAZ, implying that Hippo inhibition requires tonic YAP/TAZ activation, such as that provided by FERMT2–FAK signaling. Moreover, the overexpression of LATS1 further suppresses YAP/TAZ activity in FERMT2-depleted cells, suggesting that Hippo and integrin–FAK signaling operate as parallel, converging inputs. Thus, FERMT2 and the Hippo cascade modulate YAP/TAZ through distinct mechanisms.

These findings, by positioning FERMT2/FAK upstream of YAP/TAZ in a manner that is epistatic to Hippo, are reminiscent of mechanotransduction pathways where cytoskeletal tension sustains YAP/TAZ activity and overrides Hippo restraint [10, 17, 70]. A key question emerging from this work is how integrin/FERMT2/FAK signaling integrates with mechanical cues to control YAP/TAZ dynamics. Investigating this interplay will be essential to understanding the full implications of YAP/TAZ modulation in response to molecular and mechanical inputs. Our data indicate partial epistasis of FERMT2 over actin polymerization based mechano-transducers, thus supporting that, at least in part, FERMT2 controls YAP/TAZ independently of actin mediated mechanosignaling (hence the partial epistasis). Yet, in conditions of extreme cytoskeletal relaxation driven by anchorage independent growth, the relative contribution of FERMT2 to YAP/TAZ activity is greatly reduced, possibly implying that, while FERMT2 is not a canonical direct mechano-transducer, it may still be engaged by alternative mechanosignalling pathways, as those involving tubulin based actin nucleation [71]. Further assessment in normal and cancer cells will be necessary to clarify the potential role of FERMT2 in mechanosignaling to regulate YAP/TAZ activity.

Altogether, our results support a model in which YAP/TAZ activation is driven by integrin/FERMT2/FAK signaling, with Hippo acting as a parallel inhibitory input. We propose that feedforward and feedback mechanisms between these axes help fine-tune YAP/TAZ dynamics. Future studies should explore how mechanical and molecular signals integrate through these pathways to regulate YAP/TAZ activity in both physiological and pathological contexts.

## Methods

### Cell culture

MDA-MB-231 cells were cultured in DMEM supplemented with 10% North American serum and 1% penicillin-streptomycin (cat. no. P4333, Merck). SUM159PT cells were maintained in Ham’s F12-K medium supplemented with 5% South American serum, 1% penicillin-streptomycin, 5 µg/ml insulin, 1 µg/ml hydrocortisone, and 10 mM HEPES. MCF10DCIS cells were cultured in a 1:1 mix of DMEM:F12-K supplemented with 5% horse serum, 20 ng/ml hEGF, 10 µg/ml insulin, 0.5 µg/ml hydrocortisone, and 1% penicillin-streptomycin. MCF10A cells were cultured in the same medium with the addition of 50 ng/ml cholera toxin. HEK293 cells were cultured in DMEM containing 10% South American serum and 1% penicillin-streptomycin. hTERT RPE-1 cells were maintained in a 1:1 DMEM:F12-K medium with 10% South American serum and 1% penicillin-streptomycin. All cell lines were authenticated and verified to be mycoplasma-free by the Tissue Culture Facility at the European Institute of Oncology (https://www.research.ieo.it/research-and-technology/technological-units/cell-culture-unit-ccu/).

Long-term paclitaxel-resistant SUM159PT are a derivative of SUM159PT (paclitaxel IC50 = 8 nM) with increased tolerance to prolonged treatment with paclitaxel. Their IC50 for paclitaxel is 143 nM [45].

For screening experiments, MDA-MB-231, SUM159PT, and MCF10DCIS cell lines were transduced with lentiviruses encoding either the pCas9-p2a-BFP-p2a-Blast vector (for stable Cas9 expression) or sequentially with pMSCV-rtTA3-PGK-Puro and pRRL-TRE3G-Cas9-p2a-GFP (for inducible Cas9 expression under a tet-ON system). Selection was carried out using blasticidin and puromycin at the following concentrations, depending on cell line and resistance markers: 10 µg/ml blasticidin and 2.5 µg/ml puromycin for MDA-MB-231; 10 µg/ml blasticidin and 2 µg/ml puromycin for SUM159PT; and 2 µg/ml blasticidin and 0.5 µg/ml puromycin for MCF10DCIS. The Cas9 expression, in the tet-ON system, was induced by supplementing the culture medium with 500 ng/ml doxycycline. For 3D spheroid cultures, cells were seeded at a density of 2,000 cells/cm² in Poly(2-HEMA)-coated plates (cat. no. 09689, Polysciences) to prevent adhesion. Cells were cultured in Mammary Epithelial Cell Growth Basal Medium (MEBM; cat. no. CC-3151, Lonza), supplemented with 5 µg/ml insulin, 0.5 µg/ml hydrocortisone, 1% penicillin-streptomycin, 5 µg/ml heparin, 1X B27, 20 ng/ml hEGF, and 20 ng/ml bFGF. Spheroids were passaged every 7 days by centrifugation (350 g, 5 min), followed by trypsinization for 5 min at 37°C. Cells were counted and reseeded at the initial density for subsequent passages.

### CRISPR knockout library design and cloning

A custom CRISPR library was designed to target approximately 200 human genes previously identified as TAZ activity modulators. Guide RNA (sgRNA) selection was based on the published Brunello library [72] and the Broad Institute’s CRISPick tool (https://portals.broadinstitute.org/gppx/crispick/public), resulting in ~2000 sgRNAs. These were split into 17 barcoded sub-pools, comprising 7 sgRNAs per gene for putative TAZ activators/inhibitors, control pools including 49 non-targeting sgRNAs, 4 sgRNAs each for 12 essential genes, and 4 sgRNAs per gene for 13 known Hippo pathway regulators.

Oligos were synthesized by TWIST Bioscience as a Tier 1 ssDNA oligo pool (up to 2000 oligos, max 120 nt). Each oligo included[1]: a universal forward priming site with a BsmBI site[2], the 20 nt sgRNA sequence[3], a second BsmBI site for cloning, and [4] a sub-pool-specific reverse priming site. The complete oligo sequence was structured as follows: 5′–TGCTG–[Forward Primer: TTGACAGTGAGCG*CGTCT*CTCACC]–[sgRNA]––[Reverse Primer: GTTTGG*AGACG*]–CAC–3′ (with BsmBI sites underlined in italics). Oligos were resuspended at 0.05 ng/µl and PCR-amplified. Amplified products were purified using the QIAquick PCR Purification Kit (cat. no. 28106, Qiagen), digested with BsmBI (cat. no. R0580L, NEB), and cloned into a pre-digested, dephosphorylated pU6-sgRNA-EF1a-Thy1.1 vector via a single-step digestion-ligation reaction. Ligation products were phenol-extracted: 600 µl of a 1:1 water/phenol (pH 8) mix was added, mixed, and spun in Phase Lock Gel tubes (5Prime, QuantaBio). The aqueous phase was recovered, precipitated with 750 µl ice-cold 96% ethanol, 25 µl 3 M NaOAc (pH 5.2), and 1 µl Pellet Paint (cat. no. 69049-3, Novagen), then incubated at –80°C for 1 hour. DNA was pelleted by centrifugation ( > 15,000 rpm, 25 min, 4°C), washed with 70% ethanol, and dried. Libraries were resuspended in 6 µl water. Transformation in electrocompetent cells was then performed: 2 μl of the ligated libraries were mixed with 30 μl of MegaX DH10B T1 electrocompetent cells (cat. no. C6400-03, Invitrogen), incubated on ice for 5 minutes, then transferred to chilled cuvette (cat. no. P41050, Invitrogen). Electroporation was carried out using the following conditions: 2.0 kV, 200 Ω, 25 μF for 4-5 sec. Cells were recovered in 400 µl LB for 1 h at 32°C under shaking (225 rpm). Dilutions (1:100–1:100,000) were plated on 150 mm plates for transformation efficiency and library coverage assessment; the rest was plated undiluted across three 150 mm plates. Colonies were counted the next day. Bacteria from plates were scraped and cultured in 300 ml LB-Amp overnight. Plasmid DNA was extracted using the NucleoBond® Xtra Maxi Kit (Macherey-Nagel) and validated by sequencing for sgRNA representation.

### Lentiviral library production and titration

To produce the lentiviral library, HEK293T cells were seeded in 100 mm dishes with DMEM supplemented with 10% South American serum and cultured overnight to reach 70–80% confluence at the time of transfection. Transfection was carried out using Lipofectamine 3000 (Thermo Fisher Scientific). Two solutions were prepared: a mix containing 20 µl Lipofectamine 3000 in 480 µl Opti-MEM, and a second one with 20 µl P3000 reagent, 5 µg pPAX2, 5 µg pMD2.G, and 10 µg of the library DNA in 500 µl Opti-MEM. The DNA mix was transferred to the Lipofectamine solution and incubated at R.T. for 15 min. The transfection mixture was then added dropwise to the cell culture dish. After 6 hours, the medium was replaced with fresh DMEM. Viral supernatants were collected at 24, 32, and 48 hours post-transfection, filtered through a 0.45 µm membrane, and stored at –80°C. Titration of the viral library was performed via FACS on sgRNA-transduced cells, using the Thy1.1 surface marker. Cells were transduced with serial dilutions of viral supernatant in the presence of polybrene. At 48 hours post-transduction, cells were stained with APC-conjugated anti-Thy1.1 antibody (cat. no. 17-0900-82, eBioscience; 1:200 dilution) in staining buffer (0.5% BSA, 0.5 mM EDTA in PBS). The percentage of Thy1.1-positive cells was plotted against viral volume to determine titer.

### In vitro CRISPR-KO screen

For 2D screens, cells were transduced at low multiplicity of infection (MOI) to ensure 500× library coverage, which was maintained throughout the experiment. 48 hours post-transduction, Thy1.1 expression was analyzed by FACS to confirm transduction efficiency. A sample was collected as time point zero (T0) for genomic DNA (gDNA) isolation, and three biological replicates were propagated per cell line. Selection was performed with G418, and post-selection (PS) samples were harvested and verified for Thy1.1 expression. SUM159PT-rtTA-Cas9-GFP cells were treated with 500 ng/ml doxycycline every 48 h during G418 selection to induce Cas9 expression. After 10 days of doxycycline treatment, GFP+ cells were FACS-sorted to exclude unresponsive cells. Sorted populations were then cultured without doxycycline. At 21 days post-transduction, cells were collected for gDNA extraction. For the 3D spheroid screen, cells were transduced with three library pools (Pool 1 and 2: 850 sgRNAs each; Pool 3: 350 sgRNAs) to account for the limited clonal efficiency in spheroid culture. Following selection, cells were seeded in 150 mm dishes at 2,000 cells/cm² under 3D spheroid culture conditions. Doxycycline was administered as in 2D cultures, with sorting of GFP+ cells following complete selection for SUM159PT-rtTA-Cas9-GFP cell line. Doxycycline was re-administered during the first spheroid passage. Spheroids were cultured for four passages (each 7 days), and samples were collected at each passage for gDNA isolation.

### In vivo passive barcoding and screening

Passive barcoding was conducted in parental MDA-MB-231 cells (lacking Cas9) transduced with 300 sgRNAs. 5×10⁵ cells per mouse were injected into six NSG mice (NOD.Cg-*Prkdc*^*scid*^ *IL2rg*^*tm1Wjl*^/ SzJ, also known as NOD/SCID/IL2Rγc^−/−^). sgRNA representation in tumors was assessed by comparing pre-injection libraries to those recovered from xenografts. For in vivo screening, MDA-MB-231-Cas9-BFP cells were transduced with Pools 1, 2 (850 sgRNAs each), and 3 (300 sgRNAs) at MOI 0.1 and selected with 850 µg/ml G418 for 10 days. After selection, a PS sample was collected for gDNA isolation. The remaining cells were prepared for injection: 5×10⁵ cells per mouse were centrifuged at 200 g for 5 min and resuspended in 14 µl PBS. Matrigel (6 µl) was added for a final injection volume of 20 µl (30% Matrigel). Cells were injected orthotopically (intra-nipple) into 6 mice (Pool 3) or 15 mice (Pools 1 and 2). Tumor granules were usually palpable at day 7 post-injection. Tumor growth was monitored until reaching 1 cm in diameter. Tumor volume was calculated as (length × width²)/2, and tumors were resected at endpoint.

### Genomic DNA extraction and NGS library preparation

Genomic DNA (gDNA) was extracted using the NucleoSpin Tissue Kit (cat. no. 740952.250, Macherey-Nagel) according to the manufacturer’s protocol and quantified using the Nanodrop spectrophotometer or the Qubit dsDNA HS Assay Kit. Library amplification was carried out by a two-step PCR, which allowed the addition of 10 bp sample-barcode, specific for each sample, and sequencing adapters. The first PCR was performed using the following program: 95 °C/10 min, 29 cycles of 95 °C/30 s, 57 °C/45 s, 72 °C/30 s and 72 °C/7 min (primers are listed in supplementary materials). The PCR amplicons for each sample were then pooled, purified using the QIAquick PCR Purification Kit, quantified and the correct amplification of the expected 405-407 bp band was checked on a 2% agarose gel. The pooled DNA was used for the second round of PCR, which was performed with 10 ng of template DNA/reaction and the following program: 95 °C/10 min, 14 cycles of 95 °C/30 s, 57 °C/45 s, 72 °C/30 s and 72 °C/7 min. PCR primers are listed in supplementary materials. PCR products for each sample were pooled, purified with the QIAquick PCR Purification Kit and the correct band size was selected with AMPure XP Beads (0.7x, Agencourt). The presence of a shift between the first and the second PCR product, due to the insertion of a 42 bp fragment in the second reaction, was verified by electrophoresis on a 3% agarose gel. As a further control, samples were run on a DNA HS Bioanalyzer (Agilent) before sequencing.

### CRISPR-Cas9 knockout and shRNA constructs

sgRNAs were cloned into the lentiviral vector pU6-sgRNA-EF1a-Thy1.1. Guide sequences were selected from the screening library and are listed in supplementary materials. Plasmids were purified using the NucleoSpin Plasmid (No Lid) Mini Kit (cat. no. 740499, Macherey-Nagel), and correct insertions were confirmed by Sanger sequencing. Doxycycline-inducible shRNAs targeting FERMT2 (ULTRA-3197962 and ULTRA-3197963, pZIP-TRE3G-ZsGreen-Blasticidin constructs) and a non-targeting control (TLNSU4501) were obtained from shERWOOD (Transomic). Lentiviral particles were produced using Lipofectamine 3000, as previously described. MDA-MB-231 cells were transduced in the presence of polybrene (8 µg/ml) and selected with 10 µg/ml blasticidin.

### Competition assays

Competition assays were performed under both 2D (adherent) and 3D (non-adherent) conditions. Cas9^+^/Thy1.1^–^ (control) and Cas9^+^/Thy1.1^+^ (sgRNA-expressing) cells were mixed at a 1:1 ratio and plated under the respective conditions. Initial transduction efficiency was verified by staining with APC-conjugated anti-Thy1.1 (cat. no. 17-0900-82, eBioscience). FACS analysis was conducted every 2–3 days for 2D cultures and every 7 days across 3-4 passages in 3D cultures.

### RT-qPCR

Total RNA was isolated using the QIAGEN RNeasy® Mini Kit (cat. no. 74904), the QIAGEN RNeasy® Midi Kit (cat. no. 75142), or the Quick-RNA MiniPrep Kit (Zymo, cat. no. R1055), following manufacturers’ protocols. cDNA was synthesized using the ImProm-II Reverse Transcription System (Promega, cat. no. A3800). RT-qPCR was performed with SsoAdvanced Universal SYBR Green Supermix (Bio-Rad, cat. no. 1725274) on Bio-Rad CFX96 or CFX384 instruments. Primer sequences are provided in supplementary materials.

### Total protein extraction and western blotting

Cells were washed with cold PBS and lysed on ice using RIPA buffer supplemented with 1 mM DTT, 1 mM PMSF, 4 mM Na₃VO₄, protease inhibitors (cOmplete Mini, Roche), and phosphatase inhibitors (PhosSTOP, Roche). After 30 min of incubation at 4 °C under rotation, lysates were sonicated (10 cycles, 30 s ON/OFF, Bioraptor Plus, Diagenode) and centrifuged at maximum speed for 10 min at 4°C. Protein concentration was determined with the Pierce™ BCA Protein Assay Kit (Thermo Fisher, cat. no. 23225).

Samples were mixed with 6X Laemmli buffer (350 mM Tris-HCl pH 6.8, 30% glycerol, 10% SDS, 0.1% bromophenol blue, 6% β-mercaptoethanol), boiled at 95°C for 5 min, and resolved on 4–15% gradient TGX™ precast polyacrylamide gels (Bio-Rad). Proteins were transferred onto nitrocellulose membranes using the Trans-Blot® Turbo™ system (Bio-Rad; 30 min, 25 V, 1.0 A). Membranes were blocked with 5% BSA or milk in TBS-T, then incubated overnight at 4°C with primary antibodies (detailed below). Primary antibodies**:** YAP/TAZ 1:1000 (D24E4) (cat. no. 8418S, Cell Signaling), LATS1 1:1000 (C66B5) (cat. no. 3477, Cell Signaling), LATS2 1:1000 (D83D6) (cat. no. 5888, Cell Signaling), FERMT2 (3A3) 1:2000 (cat. no. MAB2617, Millipore), FAK 1:1000 (cat. no. 3285, Cell Signaling), Phospho-FAK (Tyr397) 1:1000 (cat. no.3283, Cell Signaling), SRC 1:1000 (cat. no. 2108S, Cell Signaling), Phospho-SRC 1:1000 (Y416) (cat. no. 2101S, Cell Signaling), Vinculin 1:500000 (cat. no. V9131, Merck), GAPDH 1:50000 (cat. no. G9545, Merck), pY391 YAP and pY444 YAP (provided by Valery’s laboratory). Membranes were then incubated with peroxidase-coupled goat anti-rabbit IgG 1:10000 (cat. no. 1706515, Bio-Rad) or goat anti-mouse IgG 1:10000 (cat. no. 1706516, Bio-Rad) for 1 h. Signal was detected with ECL substrate (cat. no. 1705061, Bio-Rad) using the ChemiDoc imaging system.

### Soft agar colony formation assay

The bottom agar layer (0.4% agar in MEBM) was dispensed into 24-well plates (500 µl/well) and allowed to solidify at 4°C for 15 min. The top layer consisted of 1,000 cells/well suspended in 0.3% agar in MEBM. After plating (500 µl/well), wells were incubated at 4°C for 15 min to solidify. Medium was added weekly to prevent desiccation. After 7–14 days, cells were stained with 0.5% Crystal Violet in 25% methanol. Images were acquired using a Nikon Eclipse Ti2 microscope (2× objective, 2×2 scan, 10% overlap). Colonies and their areas were quantified using ImageJ within a fixed region of interest (ROI).

### Cell transfection

Reverse transfection with siRNA against FERMT2 (cat. no. s21612, s21614, Silencer^TM^ Select, Life Technologies) or scrambled siRNA (Negative control No.2, cat. no. 4390847, Silencer^TM^ Select, Life Technologies) was performed using the Lipofectamine RNAiMAX Transfection Reagent. Briefly, in a 6-well plate, 12.5 nM siRNAs diluted in 500 µl Opti-MEM were incubated with 500 µl Opti-MEM containing 3.25 µl Lipofectamine RNAiMAX reagent in order to obtain 1 ml transfection mix for each well. The latter was then plated with 1 ml of resuspended cells (density of cells according to the cell line). The knock-down efficiency was verified at 48 hours from the transfection. For the rescue experiments, 24 hours post-siRNA silencing, cells were transfected with the following plasmids: pEGFP C3-LATS1 (Addgene no. 19053), pcDNA3-HA-TAZ^S89A^ (Addgene no. 32840), pQCXIH-Myc-YAP^5SA^ (Addgene no. 33093) and their respective empty vectors. The transfection was carried out using the Lipofectamine 3000 Reagent (Thermo Fisher Scientific). According to manufacturer’s protocol, Lipofectamine 3000 was diluted in 125 µl of Opti-MEM for six-well plates and mixed with an Opti-MEM solution containing 2.5 µg of DNA of interest and 3 µl P3000 Reagent. After 15 min of incubation, the transfection mix was added drop wise to the adherent cells. 6 hours post transfection, the medium was changed. Cells were harvested 48 hours post transfection for downstream analysis.

### Viral production and cell transduction

Lentivirus production was performed as described above. Cell lines of interest were transduced in presence of 8 µg/ml polybrene, washed after 6 hours and supplemented with fresh medium. 24-hours later, cells were split and seeded into antibiotic-containing medium.

For in vitro and in vivo rescue experiments, MDA-MB-231-Cas9 or SUM159PT-rtTA-Cas9 cells were transduced with lentivirus pSLIK-(tetON)-mTAZ^S89^-Hygro (tetON-TAZ^S89A^), carrying TAZ murine sequence with the amino acid substitution S89A. TAZ murine sequence was cloned in the emply pSLIK-Hygro vector (Addgene no. 25737), as described in Sberna et al. [25]. Selection was carried out as follows: 400 µg/ml in MDA-MB-231 cells and 200 µg/ml in SUM159PT cells.

### Pharmacological treatments

For betamethasone (BM) treatment (cat. no. HY-13570, MedChemExpress), MDA-MB-231 cells were serum-starved and then treated with 1 µM BM for 24 hours, based on previously published protocols [17]. FAK inhibitors treatment was performed for 24 hours on adherent cells at the following concentrations, based on previously published works [56, 59]: 0.5 µM, 1 µM and 10 µM for PND-1186 (cat.no. HY-13917, MedChemExpress); 5 µM, 10 µM and 20 µM for PF-431396 (cat.no. HY-10460, MedChemExpress).

### Cell viability and cell proliferation assays

SUM159PT and MDA-MB-231 cells were seeded in 96-well plates at a density of 1000 cells/well and treated with a dose range of paclitaxel. After 72 h of treatment, cell viability was assessed using the CellTiter-Glo Luminescent Cell Viability assay (Promega), according to the manufacturer’s protocol. Half-maximal inhibitory concentrations (IC₅₀) were calculated using nonlinear regression analysis in GraphPad Prism (GraphPad Software, Boston, MA, USA). Cell proliferation was measured using Incucyte SX5 Live-Cell Analysis system (Sartorius). Long-term paclitaxel-resistant SUM159PT cells, transfected with either FERMT2-targeting siRNA or control siRNA (siC), were seeded in 24-well plates in quadruplicate at a density of 3×10^4^ cells/well and treated with either 25 nM paclitaxel or vehicle control (DMSO). Prior to image acquisition, Propidium Iodide (PI) was added to the culture medium to a final concentration of 1ug/ml. Cell growth was monitored every day using a 4x objective for whole-well imaging from day 0 (seeding) to day 5. Analysis was carried out using the Incucyte analysis software. Data are presented as cell area confluency normalized to the initial confluence at day 0.

### Immunofluorescence and image analysis

To quantify the nuclear-to-cytoplasmic mean intensity ratio of YAP and TAZ and F-actin mean intensity, immunofluorescence was performed on hTERT RPE-1 cells seeded on glass coverslips. Cells were fixed in 4% paraformaldehyde (PFA) for 15 min, permeabilized with 0.2% Triton X-100 for 15 min, and blocked with 2% BSA for 30 min. Primary antibodies were incubated for 1 hour at RT: anti-TAZ (1:100, cat. no. 580235, BD Biosciences); anti-YAP (1:100, D8H1X, Cell Signaling). After PBS washes, secondary antibodies were added: Alexa Fluor Plus 488 (anti-mouse, cat. no. A32766) and Alexa Fluor Plus 555 (anti-rabbit, cat. no. A31572), along with Phalloidin (cat. no. AB176759, Abcam). Nuclei were stained with DAPI. Samples were mounted using Mowiol-DABCO mounting medium (9.6% MOWIOL, cat. no. 475904-100 g, Calbiochem 1.5% DABCO, cat. no. D27802, Sigma, 12% Glycerol, 0.1 M Tris HCL pH 7.4 in H2O MilliQ solution) and imaged with a Nikon CSU-W1 spinning disk confocal microscope (Nikon Europe B.V.) equipped with a L6Cc laser source (Oxxius), a multiband dichroic mirror, single-band emission filters and a Prime BSI sCMOS camera (Teledyne Photometrics). Multi-channel z-stacks (10 µm depth) were acquired using a 60X/1.4 oil immersion objective with a voxel size of 0.11×0.11×0.30 μm^3^. Image analysis was conducted using a custom Python-based pipeline comprising (i) z-stack maximum intensity projection, (ii) cell and nuclear segmentation (using Phalloidin and DAPI signals) via the deep learning algorithm Cellpose [73], (iii) assignment of nuclei to corresponding cells, (iv) quantification of mean intensity for F-actin and extraction of mean nuclear and cytoplasmic intensities for both TAZ and YAP signals. Nuclear-to-cytoplasmic mean intensity ratios were then computed for each cell. Image analyses were performed on 3 biological replicas, each consisting of at least 200 cells.

### In vivo studies

In vivo experiments were performed on 10–12 week-old NSG female mice. MDA-MB-231 Cas9-BFP cells (5×10⁵) were injected intra-nipple as described above. Tumor growth was monitored two to three times/week using caliper. For rescue experiments in FERMT2 knock-out tumor cells, mice were fed doxycycline-containing food starting 72 hours prior to injection, either for the entire experiment (durable rescue) or for a limited time (transient rescue). For FERMT2 silencing with inducible shRNAs, doxycycline food was introduced at the onset of tumor exponential growth. All methods were performed in accordance with the relevant guidelines and regulations.

### Bioinformatic analysis

#### In vitro screening

Data analysis was performed using MAGeCK [27]. For each gene, log2 fold change (β-score) and *p*-values were calculated across sgRNAs. Statistical significance was defined as *p* < 0.05 and β-score <–0.6 or >0.6.

#### In vivo screening

Analysis was performed using Gene Set Enrichment Analysis (GSEA) algorithm [74]. sgRNAs with raw counts below 250 reads post-antibiotic selection (PS) were excluded from the analysis. Counts were normalized (CPM), and guide abundances in tumors were normalized against input libraries at the PS time point. Then, GSEA algorithm was applied: for each gene, the relative counts of its sgRNAs were compared to the counts of all sgRNAs for all the genes, thus determining whether a gene’s abundance was significantly increased or decreased. GSEA outputs included NES, FDR, and normalized *p*-values.

#### DepMap data analysis

The “YAP/TAZ signature” was defined from the CORDENONSI_YAP_CONSERVED _SIGNATURE v2024.1.Hs.gmt gene list from MSigDB [75], excluding cell cycle genes (WP_CELL_CYCLE.v2024.1.Hs.gmt), resulting in a final list of 53 genes. For each cell line in DepMap v20Q4, the average Z-score across these genes was calculated. Cell lines were then ranked, and the top and bottom 25% were defined as the “high” and “low” YAP/TAZ signature groups, respectively. Gene essentiality (defined as gene effect scores below -1) was calculated using DepMap v20Q4 gene effect data and assessed for each gene across high- and low-YAP/TAZ signature cell lines. Fisher’s exact test was then applied to identify differentially essential genes between the two groups (adjusted *p*-value < 0.05). Gene ontology analysis was conducted using custom R functions, with the MSigDB C5 collection as reference database.

### Ethics approval and consent to participate

All animal procedures followed Italian guidelines (D.L.vo 26/14 and following amendments), in accordance with EU directive 2010/63, and were approved by the OPBA and the Ministry of Health (142/2020-PR).

## Supplementary information

Supplementary figures

Supplementary materials

dataset 1

dataset 2

dataset 3

dataset 4

uncropped WB

raw data

## Data Availability

Data are included in the Supplementary Data files. Data and scripts are available upon request.
