# Supplementary material for: Functional genomic screens uncover FERMT2 as a critical regulator of YAP/TAZ-driven tumorigenicity
Source: Cell Death Differ. 2026 Mar 6;33(9):1907–22. doi: 10.1038/s41418-026-01694-w (PMC13545242; doi:10.1038/s41418-026-01694-w)
Supplement: Supplementary file 1 [file 41418_2026_1694_MOESM2_ESM.pdf]

List of antibodies

ANTIBODY	BRAND	CODE	APPLICATION
YAP/TAZ (D24E4)	Cell signaling	8418S	IB
LATS1 (C66B5)	Cell signaling	3477	IB
LATS2 (D83D6)	Cell signaling	5888	IB
FERMT2 (3A3)	Millipore	MAB2617	IB
FAK	Cell signaling	3285	IB
Phospho-FAK (Tyr397)	Cell signaling	3283	IB
Phospho-SRC (Y416)	Cell signaling	2101S	IB
Vinculin	Merck	V9131	IB
GAPDH	Merck	G9545	IB
YAP (D8H1X)	Cell signaling	14074S	IF
TAZ (M2-616)	BD	580235	IF
CD90.1 (Thy-1.1) (HIS51)	eBioScience	17-0900-82	FACS

List of primers

PRIMER	SEQUENCE	APPLICATION
Universal Forward Primer	TTGACAGTGAGCGCGTCTCTCACC	universal forward primer for sub-library amplification
Library 1	TACCGCAGCCATGAAGAACC	reverse primer sequence for sub-library amplification
Library 2	GCTGCTGTCTAAGGCACAGG	reverse primer sequence for sub-library amplification
Library 3	TACTTGGCAGTACATCTAGG	reverse primer sequence for sub-library amplification
Library 4	GCCTCAGCCACCTACTACAG	reverse primer sequence for sub-library amplification
Library 5	CTGCAAGGCCTCCAGGTTGG	reverse primer sequence for sub-library amplification
Library 6	TTGAACCTCCTCGTTCGACC	reverse primer sequence for sub-library amplification
Library 7	ATTCTGCACGCTTCAAAGC	reverse primer sequence for sub-library amplification
Library 8	AGACCAAAGGTGTCTTCCAC	reverse primer sequence for sub-library amplification
Library 9	CGCAGTGATGGTGGTGATGG	reverse primer sequence for sub-library amplification
Library 10	GCTCCCGGAAAGTAAGGACG	reverse primer sequence for sub-library amplification
Library 11	GTGAAGACACAGCTGACCTC	reverse primer sequence for sub-library amplification
Library 12	GACATGCTCCCAGCCGATGC	reverse primer sequence for sub-library amplification
Library 13	CACACACGTGGGCTCCTTCG	reverse primer sequence for sub-library amplification
Library 14	CCACGGGTTCGTTCCACAGG	reverse primer sequence for sub-library amplification
Library 15	GATCCACAATGGCACCGTGC	reverse primer sequence for sub-library amplification
Library 16	TCAGACCCCCACGGGCATGC	reverse primer sequence for sub-library amplification
Library 17	TCCTACAACTCCGGAGCAGT	reverse primer sequence for sub-library amplification
sgDeepSeq_p7_Nras	GCATACGAGATAGCTAGCCACC	forward primer - first PCR reaction
sgDeepSeq_rev GAAG	CTCTTTCCCTACACGACGCTCTTCCGATCTCATAGCCTTCTTCCAGCATAGCTCTTAAAC	reverse primer - first PCR reaction
sgDeepSeq_rev AGCT	CTCTTTCCCTACACGACGCTCTTCCGATCTTCCAGCAGCTTTCCAGCATAGCTCTTAAAC	reverse primer - first PCR reaction
sgDeepSeq_rev CAGA	CTCTTTCCCTACACGACGCTCTTCCGATCTTTACGCTCTGTTCCAGCATAGCTCTTAAAC	reverse primer - first PCR reaction
sgDeepSeq_rev CAAC	CTCTTTCCCTACACGACGCTCTTCCGATCTTTGAATGTTGTTCCAGCATAGCTCTTAAAC	reverse primer - first PCR reaction
sgDeepSeq_rev TCGA	CTCTTTCCCTACACGACGCTCTTCCGATCTTTAATCTAGGTTCCAGCATAGCTCTTAAAC	reverse primer - first PCR reaction
sgDeepSeq_rev TAGG	CTCTTTCCCTACACGACGCTCTTCCGATCTCTGTGGCCTATTCCAGCATAGCTCTTAAAC	reverse primer - first PCR reaction
sgDeepSeq_rev GTTC	CTCTTTCCCTACACGACGCTCTTCCGATCTTCGGAAGAACTTCCAGCATAGCTCTTAAAC	reverse primer - first PCR reaction
sgDeepSeq_rev GGAT	CTCTTTCCCTACACGACGCTCTTCCGATCTAGCAATATCCTTCCAGCATAGCTCTTAAAC	reverse primer - first PCR reaction
sgDeepSeq_rev CTCA	CTCTTTCCCTACACGACGCTCTTCCGATCTTGCTCGTGAGTTCCAGCATAGCTCTTAAAC	reverse primer - first PCR reaction
sgDeepSeq_rev CGAA	CTCTTTCCCTACACGACGCTCTTCCGATCTCGTTACTTCGTTCCAGCATAGCTCTTAAAC	reverse primer - first PCR reaction
sgDeepSeq_rev TGAG	CTCTTTCCCTACACGACGCTCTTCCGATCTCATGCTTTCGTTCCAGCATAGCTCTTAAAC	reverse primer - first PCR reaction
sgDeepSeq_rev ATCC	CTCTTTCCCTACACGACGCTCTTCCGATCTAAGACAGGATTTCCAGCATAGCTCTTAAAC	reverse primer - first PCR reaction
sgDeepSeq_rev AGGA	CTCTTTCCCTACACGACGCTCTTCCGATCTCAGCAATCCTTTCCAGCATAGCTCTTAAAC	reverse primer - first PCR reaction
sgDeepSeq_rev ACTC	CTCTTTCCCTACACGACGCTCTTCCGATCTTCCTTGGAGTTTCCAGCATAGCTCTTAAAC	reverse primer - first PCR reaction
sgDeepSeq_rev ACGT	CTCTTTCCCTACACGACGCTCTTCCGATCTAGGTTAACGTTTCCAGCATAGCTCTTAAAC	reverse primer - first PCR reaction
sgDeepSeq_rev AAGC	CTCTTTCCCTACACGACGCTCTTCCGATCTGTCATCGCTTTTCCAGCATAGCTCTTAAAC	reverse primer - first PCR reaction
sgDeepSeq_rev GAGT	CTCTTTCCCTACACGACGCTCTTCCGATCTGACCTAACTCTTCCAGCATAGCTCTTAAAC	reverse primer - first PCR reaction
sgDeepSeq_rev CTTG	CTCTTTCCCTACACGACGCTCTTCCGATCTCTACCACAAGTTCCAGCATAGCTCTTAAAC	reverse primer - first PCR reaction
sgDeepSeq_rev AGAC	CTCTTTCCCTACACGACGCTCTTCCGATCTCCAGAGGTCTTTCCAGCATAGCTCTTAAAC	reverse primer - first PCR reaction
Rev2_p5_sgDeepSeq	AATGATACGGCGACCACCGAGATCTACACTCTTTCCCTACACGACGCT	forward primer - second PCR reaction
Fwd2_p7_sgDeepSeq	CAAGCAGAAGACGGCATACGAGATAGCTAGCCACC	reverse primer - second PCR reaction
CTGF Forward	CCTGCAGGCTAGAGAAGCAG	qPCR
CTGF Reverse	TGGAGATTTTGGGAGTACGG	qPCR
CYR61 Forward	AAGAAACCCGGATTTGTGAG	qPCR
CYR61 Reverse	GCTGCATTTCTTGCCCTTT	qPCR
ANKRD1 Forward	GATCGAATTCCGTGATATGCT	qPCR
ANKRD1 Reverse	AAACATCCAGGTTTCCTCCA	qPCR
AMOTL2 Forward	AGGCTGCAGAGAGACAATGAG	qPCR
AMOTL2 Reverse	CTCAGAGAGCCGCTGGATT	qPCR
RPLP0 Forward	TTCATTGTGGGAGCAGAC	qPCR
RPLP0 Reverse	CAGCAGTTTCTCCAGAGC	qPCR
FERMT2 Forward	TACTGCTTTCACCTGCCTGAG	qPCR
FERMT2 Reverse	GAGCGGGTGGACAAGAAAATG	qPCR
GILZ1 Forward	TCTGCTTGGAGGGGATGTGG	qPCR
GILZ1 Reverse	ACTTGTGGGGATTCGGGAGC	qPCR
LATS1 Forward	AAATGAGTTACCAAGATCCTCGAC	qPCR
LATS1 Reverse	CGGTTAACTGATTGCTGCAC	qPCR
WWTR1 Forward	TATCCCAGCCAAATCTCGTG	qPCR
WWTR1 Reverse	TTCTGCTGGCTCAGGGTACT	qPCR
YAP Forward	CTCACAGCAGAACCGTTTCCC	qPCR
YAP Reverse	AGCCAAAACAGACTCCATGTCA	qPCR
CAPZB Forward	GGCTGGAGATGGATCAAAGA	qPCR
CAPZB Reverse	CCAGAGCCAGATTTGTTGGT	qPCR
CFL1 Forward	CCTACGCCACCTTTGTCAAG	qPCR
CFL1 Reverse	CTTCTTGATGGCGTCCTTGG	qPCR
GSN Forward	AGCTGGCCAAGCTCTACAAG	qPCR
GSN Reverse	TTCCTCTCCTCCGTGTTTGC	qPCR

List of sgRNAs

GENE	SEQUENCE
sgROSA26#2	CGCGGCAGGCCCTCCGAGCG
sgROSA26#3	CCCGATCCCCTACCTAGCCG
sgPOLR2D#1	TAGCACTCACCTACGAACAC
sgPOLR2D#2	TCCGCGGGCTGGCGACGTAG
sgPOLR2D#3	GCAGCAGAATGAGAGTGCAG
sgPOLR2D#4	TCACAGCTCATCTTTCCTAA
sgFERMT2#1	CACCCTGAGAGTGACCGGCG
sgFERMT2#2	AGGCGTGATGCTTAAGCTGG
sgFERMT2#3	CATCATGAGCATCATAAGTG
sgFERMT2#4	CTGAATACCATACTTATCTA
sgLATS2#1	AAGACGCCGCCGGAGACCGG
sgLATS2#2	ACCAGCAGAAGGTTAACCGG
sgLATS2#3	AGAGCCGCAAAAGCGCCAAG
sgLATS2#4	GTAGGACGCAAACGAATCGC
sgSAV1#1	ATAACACAAATACAACTCAC
sgSAV1#2	ATGCCTTCATTCATCCGGCA
sgSAV1#3	GGGGTAAATCTTCAGAACCA
sgSAV1#4	TCTCTTCTCATTATTTCATG

List of siRNAs

siRNA	Sequence 5'-3'	BRAND
siFERMT2#12	GAUCACGACUGAUAUAACUtt	Ambion_Silencer Select
siFERMT2#14	GCUUCGUUGUGACAAUGAAtt	Ambion_Silencer Select
siCapzb s2401	CGAGAUCUACUUUGGAAAAtt	Ambion_Silencer Select
siCapzb s2402	GACUUUUGCAGACAAAUCAtt	Ambion_Silencer Select
siCfl1 s2937	AGGUGUUCAACGACAUGAAtt	Ambion_Silencer Select
siCfl1 s2938	GGAUCAAGCAUGAAUUGCAtt	Ambion_Silencer Select
siGsn s6242	GGAUGAUGAUUACUGGUCUtt	Ambion_Silencer Select
siGsn s6243	GGGUUGGAAAGGAUUCUCAtt	Ambion_Silencer Select
