# Supplementary material for: Functional genomic screens uncover FERMT2 as a critical regulator of YAP/TAZ-driven tumorigenicity
Source: Cell Death Differ. 2026 Mar 6;33(9):1907–22. doi: 10.1038/s41418-026-01694-w (PMC13545242; doi:10.1038/s41418-026-01694-w)

Figure 4d

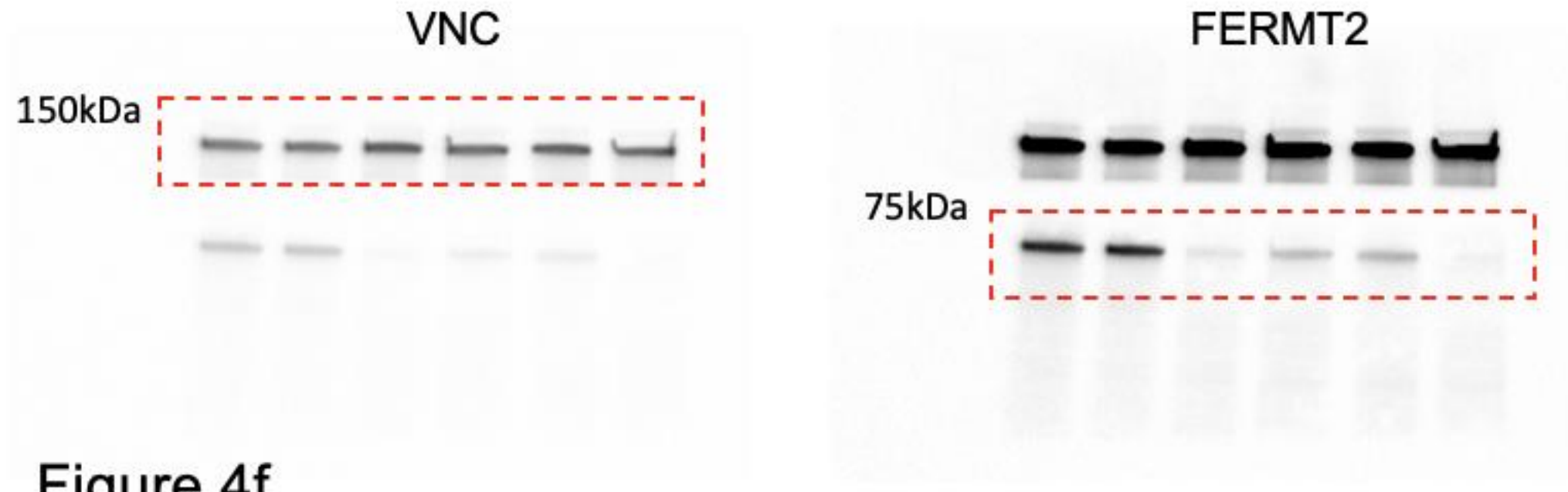

Figure 4f

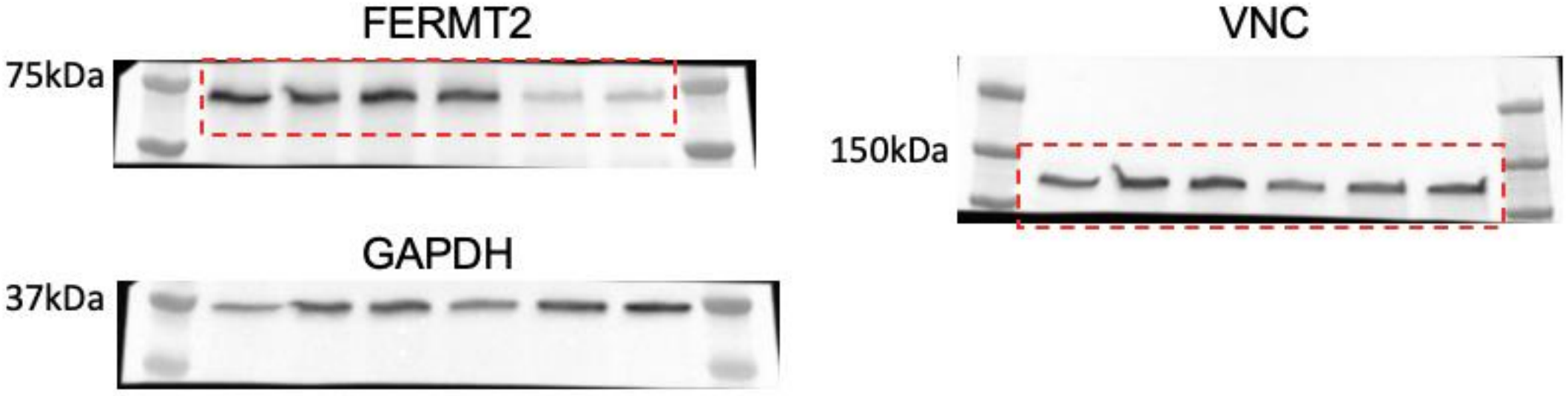

Figure 5f

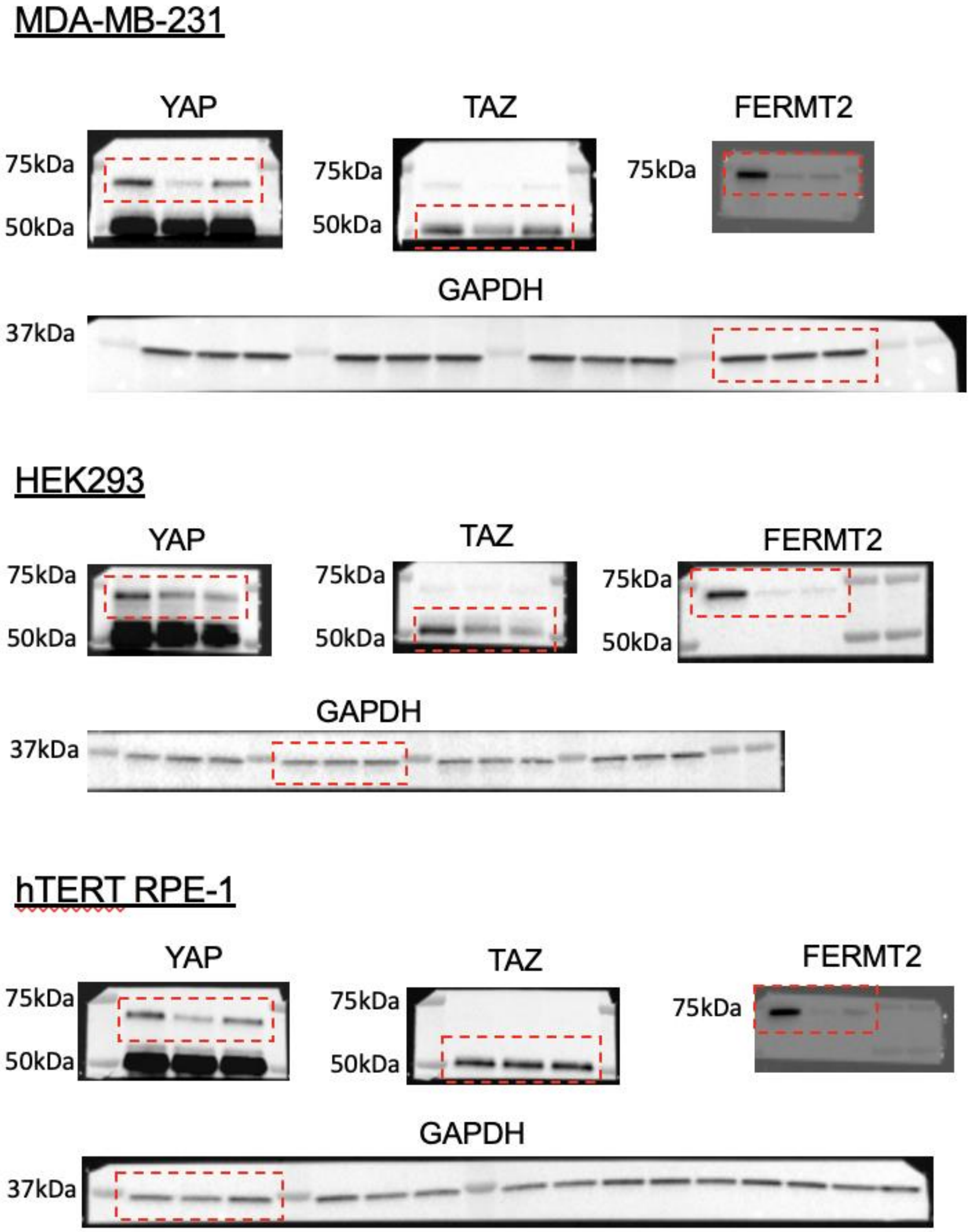

MDA-MB-231
YAP
TAZ
FERMT2
75kDa
50kDa
75kDa
50kDa
75kDa
GAPDH
37kDa
HEK293
YAP
TAZ
FERMT2
75kDa
50kDa
75kDa
50kDa
75kDa
50kDa
GAPDH
37kDa
hTERT RPE-1
YAP
TAZ
FERMT2
75kDa
50kDa
75kDa
50kDa
75kDa
GAPDH
37kDa

Figure 6a

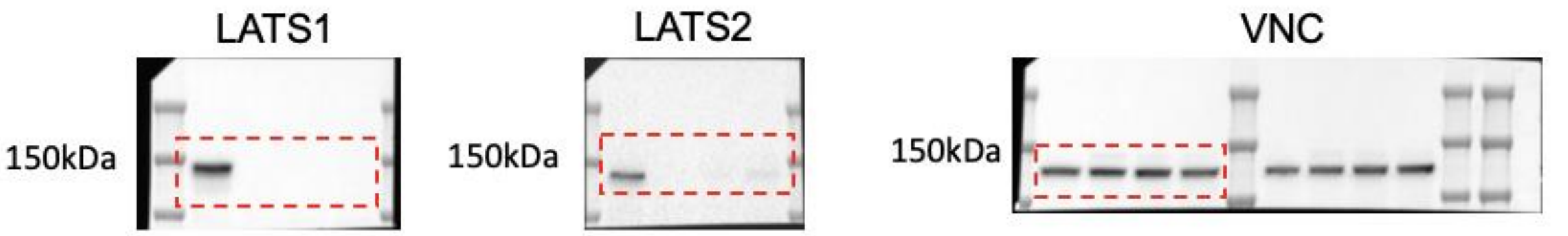

Figure 6d

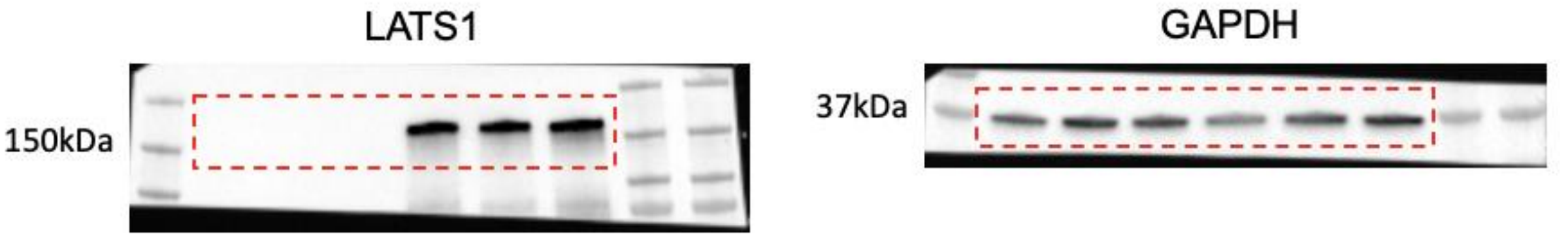

Figure 7a

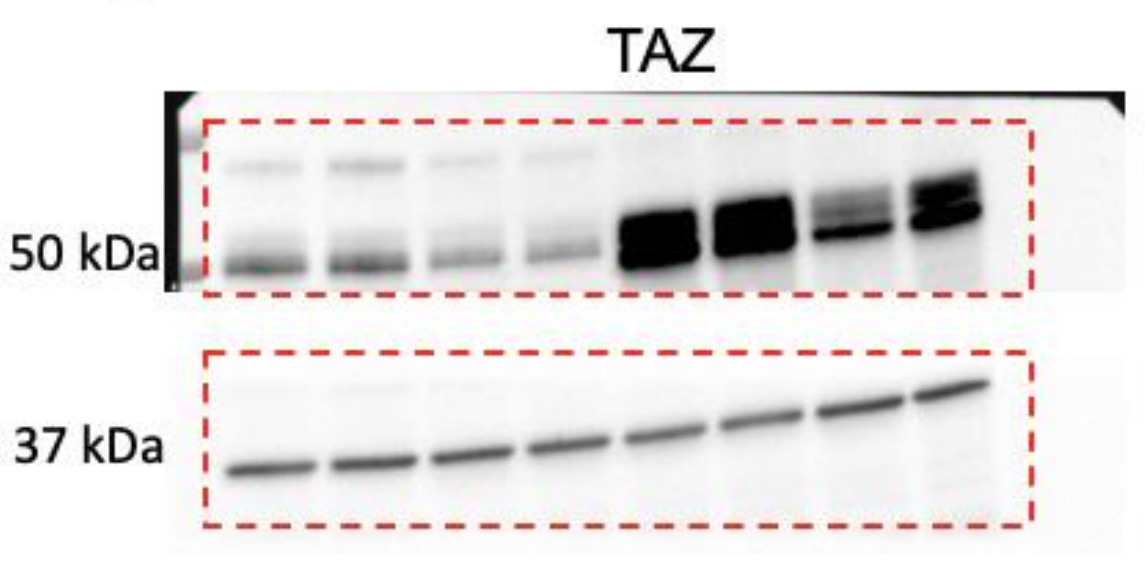

Figure 7c

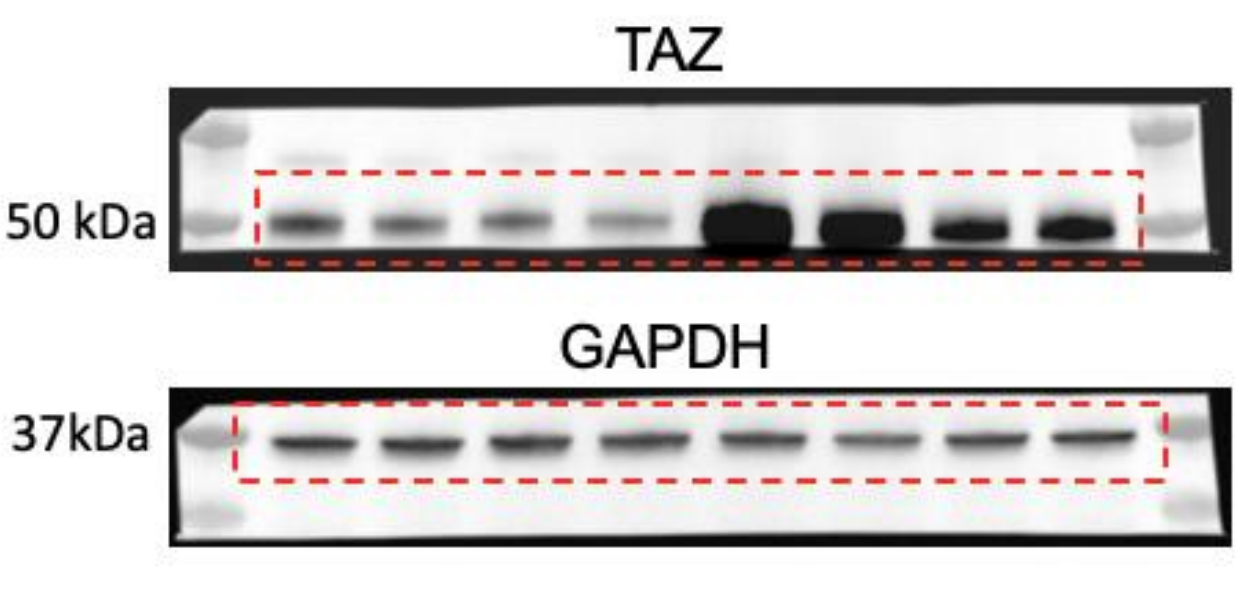

Figure 8a

<u>MDA-MB-231</u>

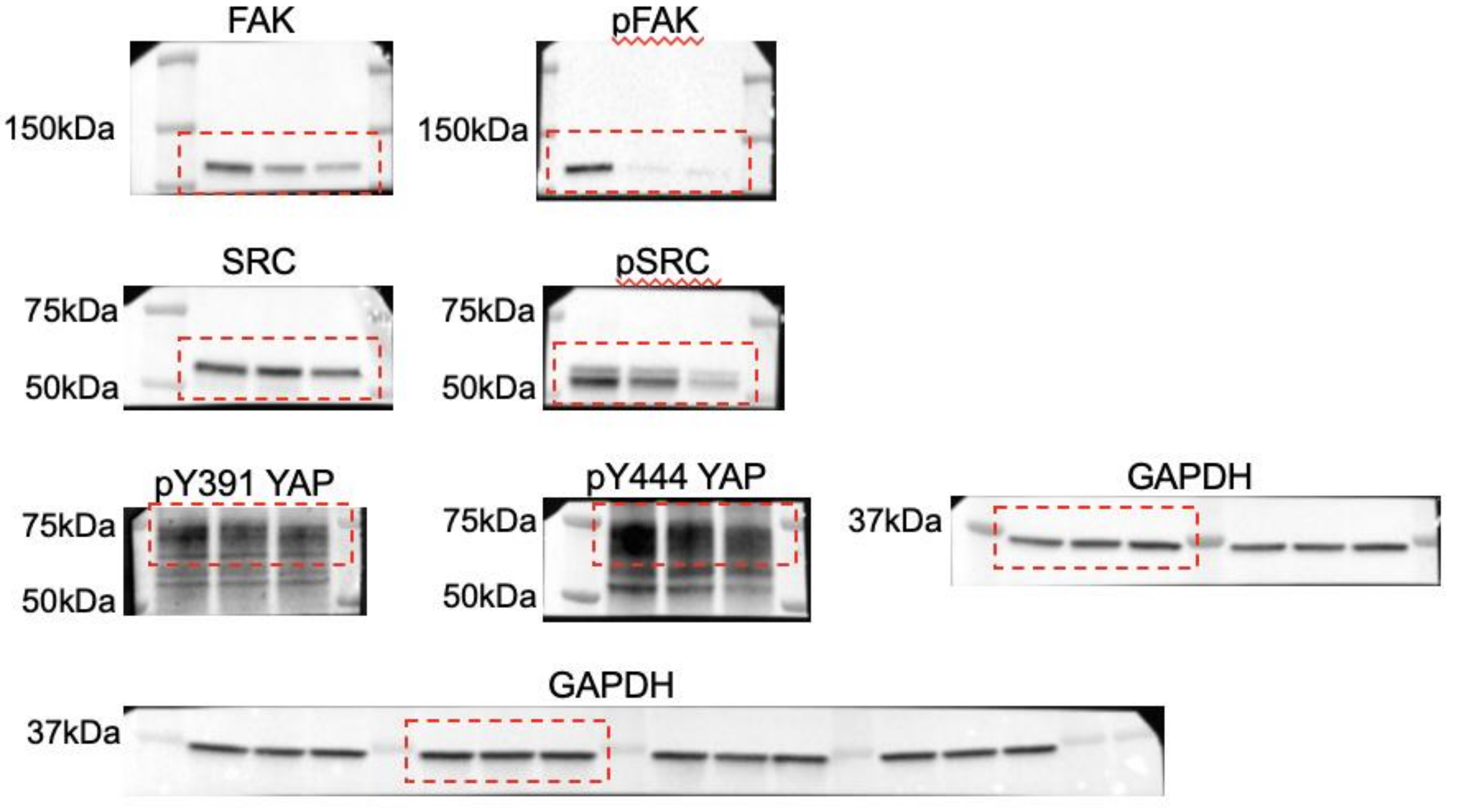

<u>HEK293</u>

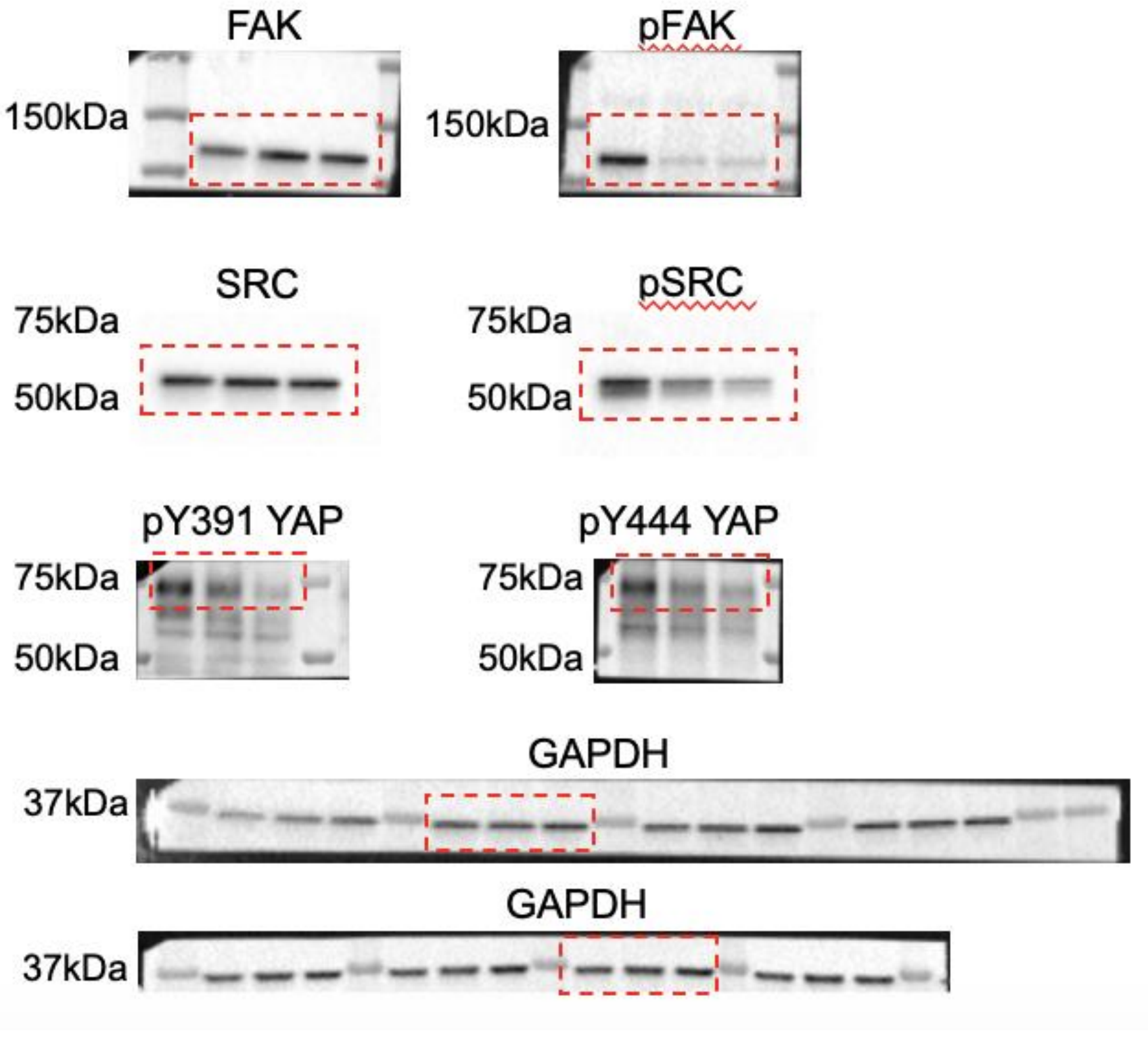

hTERT RPE-1

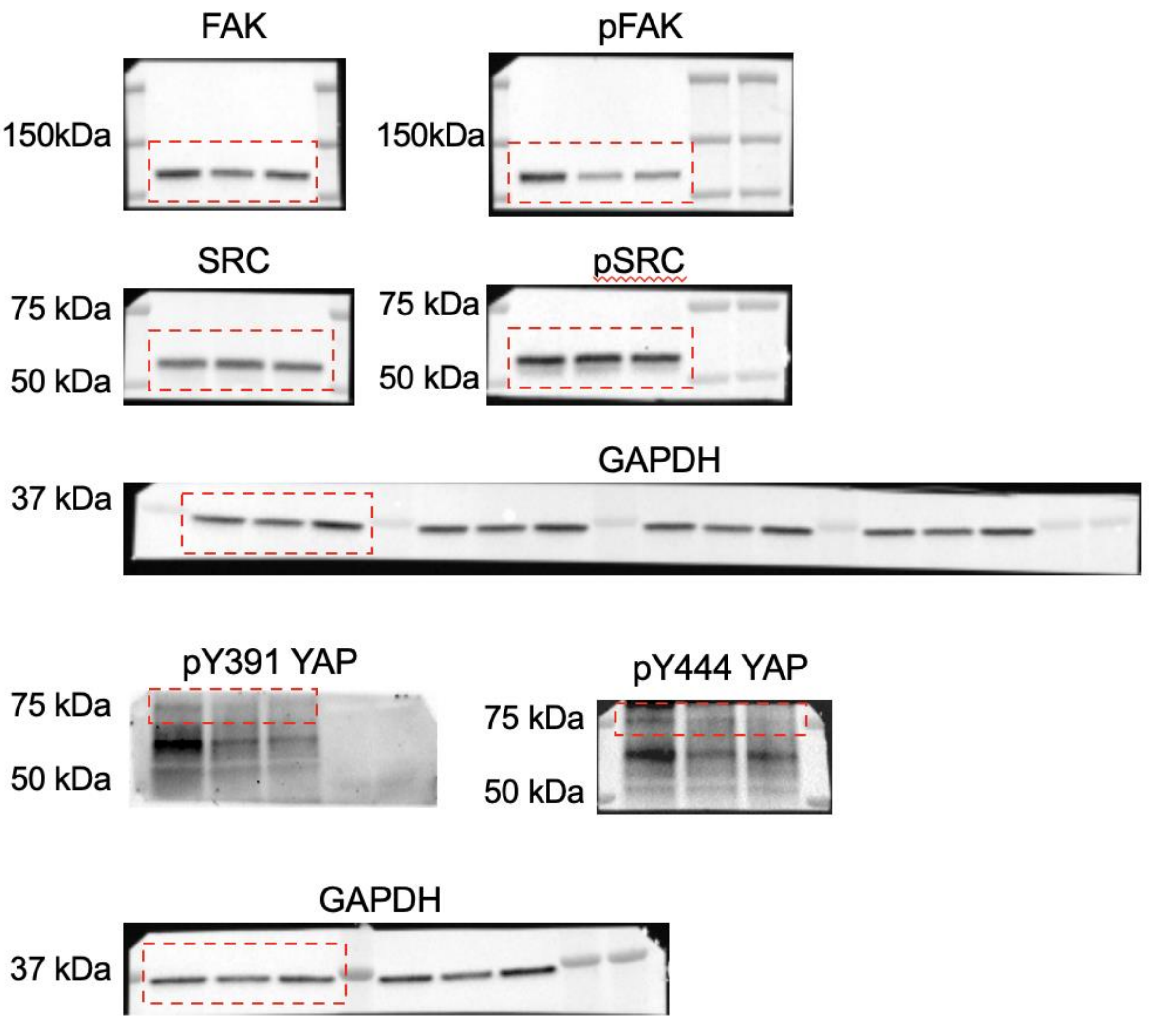
FAK
150kDa
pFAK
150kDa
SRC
75 kDa
50 kDa
pSRC
75 kDa
50 kDa
GAPDH
37 kDa
pY391 YAP
75 kDa
50 kDa
pY444 YAP
75 kDa
50 kDa
GAPDH
37 kDa

Figure 8d

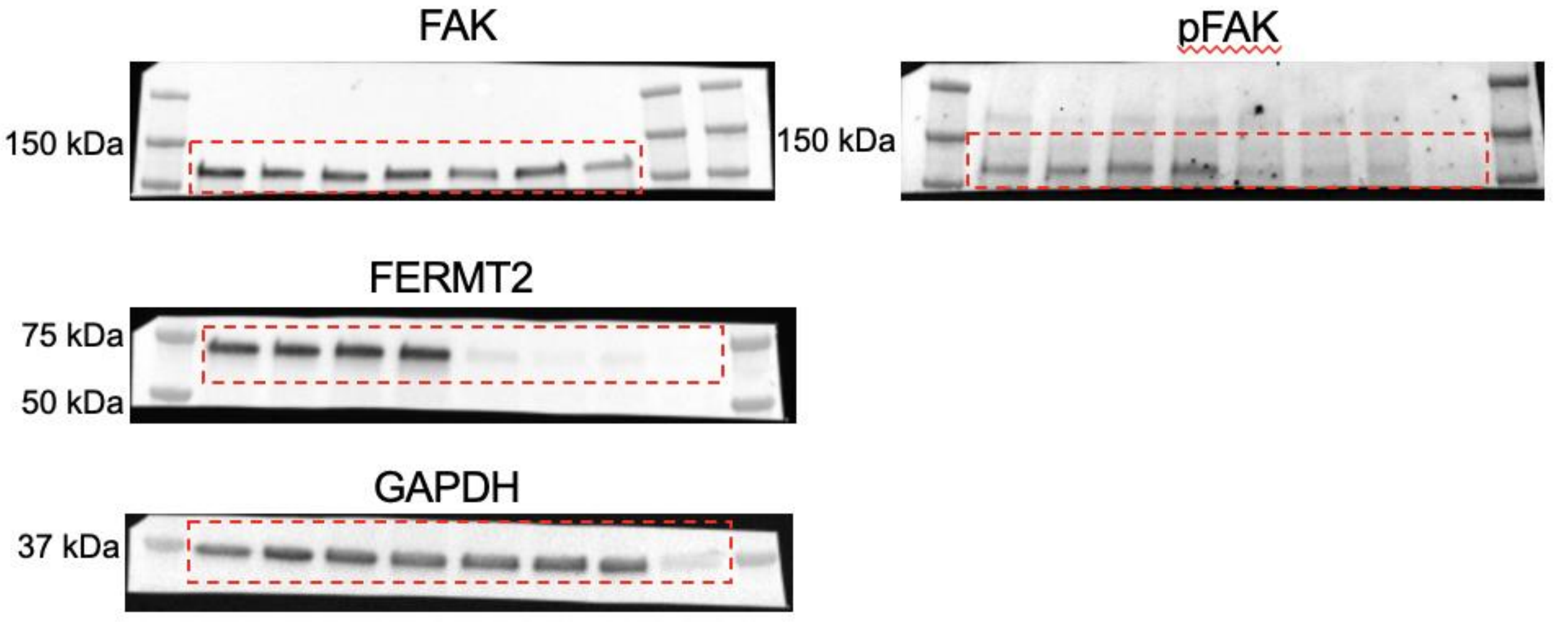

Figure 8g

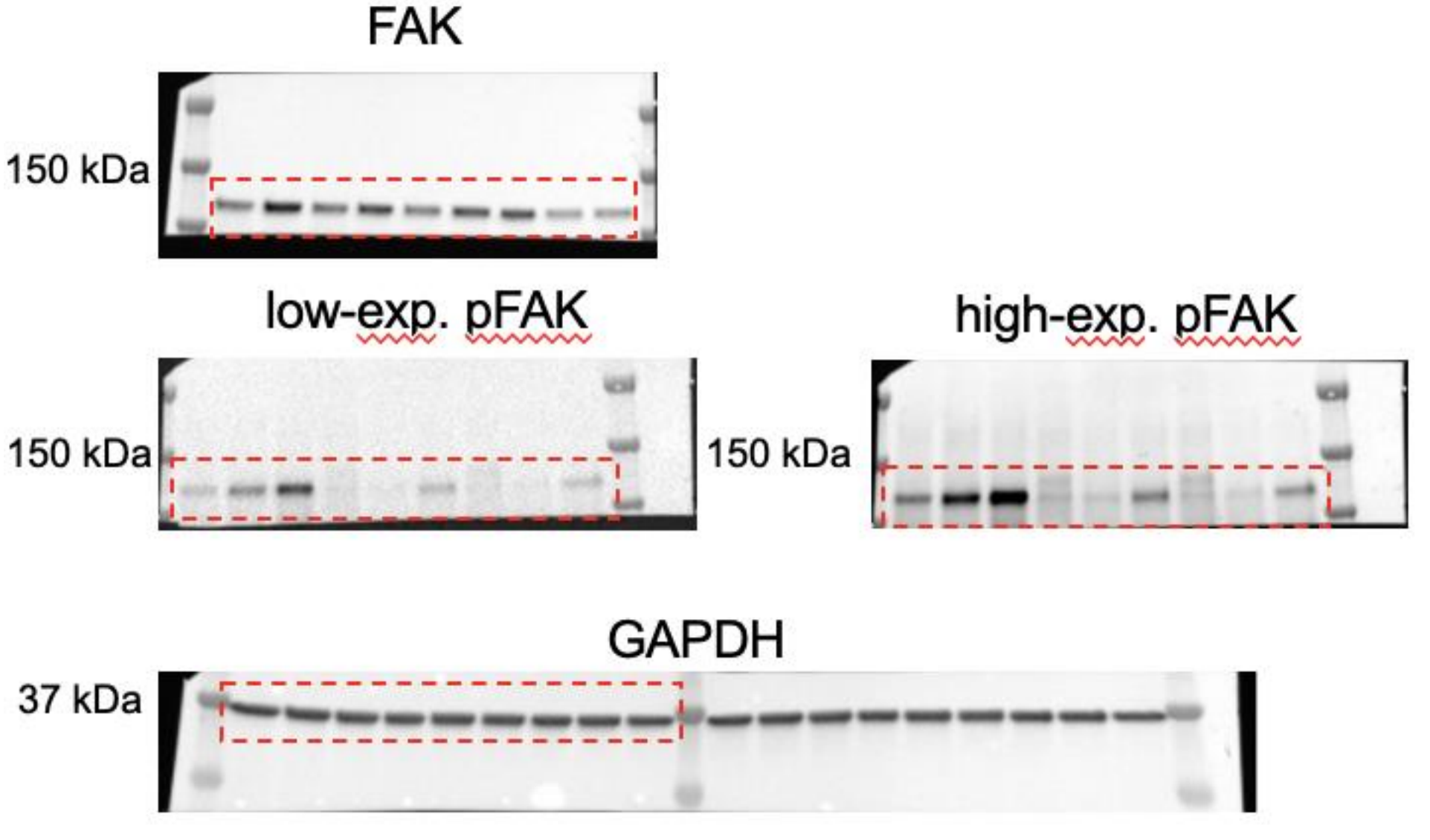

Figure S9C

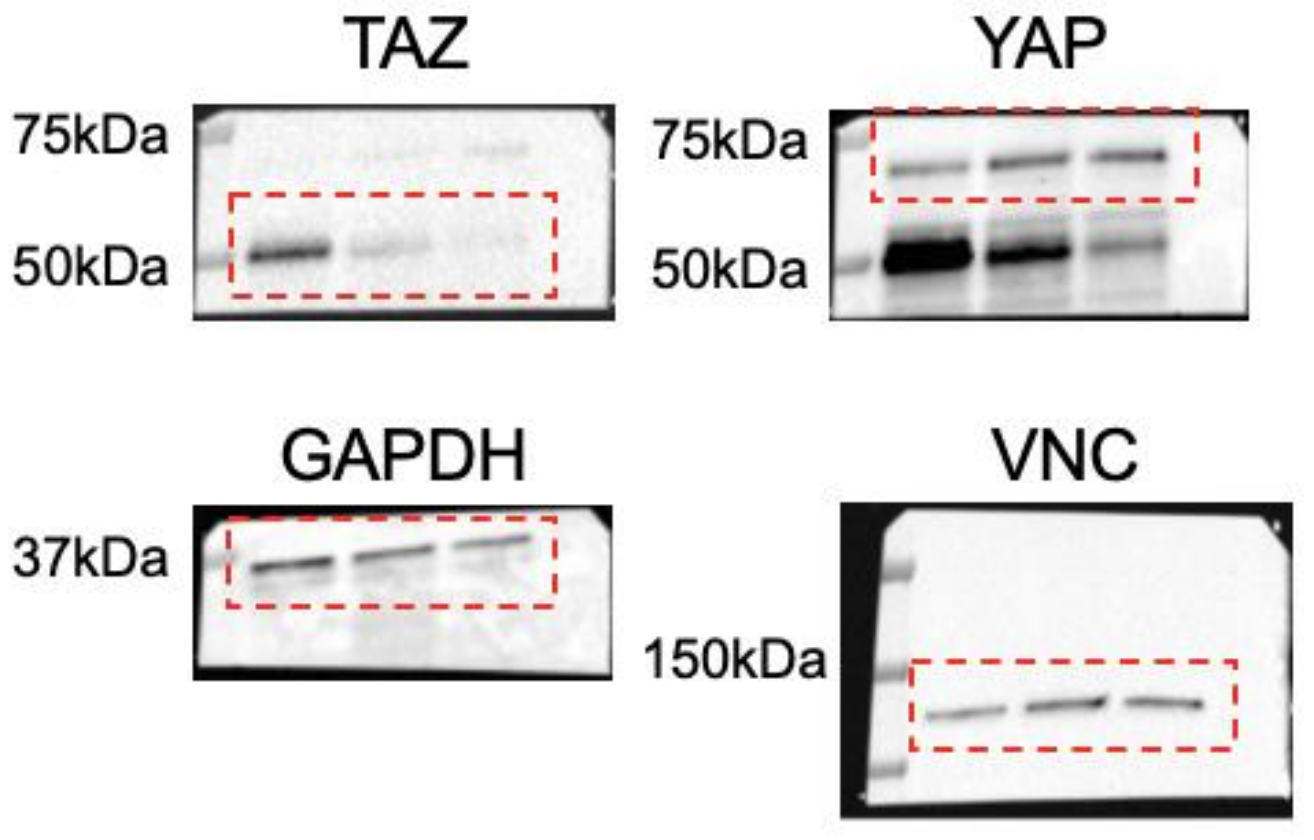

Figure S10A

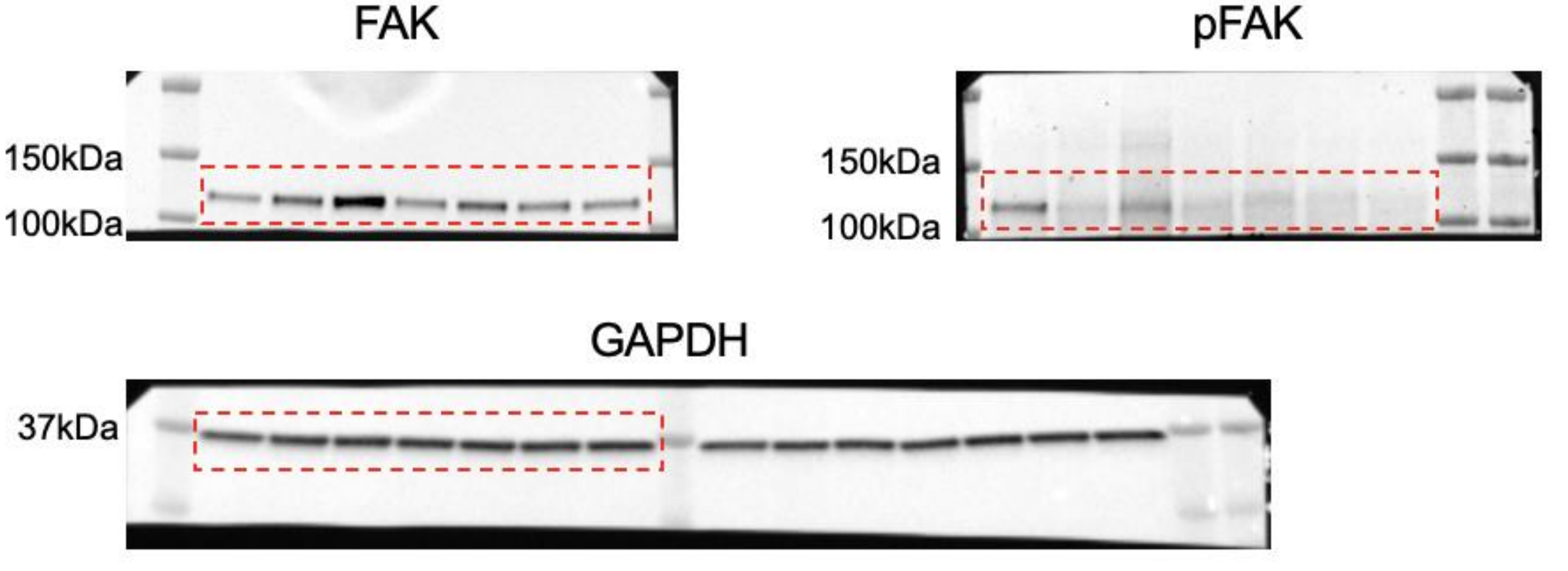

Supplement: Supplementary file 2 [file 41418_2026_1694_MOESM7_ESM.pdf]
