# Supplementary material for: Functional genomic screens uncover FERMT2 as a critical regulator of YAP/TAZ-driven tumorigenicity
Source: Cell Death Differ. 2026 Mar 6;33(9):1907–22. doi: 10.1038/s41418-026-01694-w (PMC13545242; doi:10.1038/s41418-026-01694-w)
Supplement: Supplementary file 3 [file 41418_2026_1694_MOESM1_ESM.docx]

**Supplementary figures**

**Supplementary Figure S1.** Distribution of sgRNAs in 2D (a) and 3D (b) screens. Volcano plots report the log_2_ fold change of the relative abundance of the sgRNAs detected at the end of the screen (x-axis), and the false discovery rate (FDR) calculated by MAGeCK (y-axis). sgRNAs targeting essential genes are reported in blue, while the non-targeting sgRNAs (neutral) are reported in red. Dashed lines mark significance thresholds arbitrarily set based on the distribution of neutral and essential sgRNAs.

**Supplementary Figure S2.** GSEA-based ranking analysis of sgRNAs targeting representative genes in *in vivo* screen. Top panels: Enrichment plots of the three representative types of library genes: drop-out genes (genes whose sgRNAs are depleted), drop-in genes (whose sgRNAs are enriched), and no-hit (sgRNAs not significantly changed). Bottom panels: enrichment plots for control genes.

**Supplementary Figure S3.** Correlation of the mRNA expression of FERMT2 and TAZ (WWTR1) or YAP1 in tumors (data from TCGA and cBioportal).

**Supplementary Figure S4.** Word cloud of the GO-terms associated with the genes showing co-dependence with FERMT2.

**Supplementary Figure S5.** Waterfall plot of the top 100 DepMap co-dependencies of FERMT2, TAZ (WWTR1), and YAP. Genes were ranked based on the correlation coefficient of the gene score. Common codependent genes are color-coded as follows: in red, genes codependent with both FERMT2 and TAZ (WWTR1); in blue, genes codependent with YAP and TAZ, in green, genes codependent with FERMT2, YAP, and TAZ (WWTR1); in purple, genes codependent with FERMT2 and YAP

**Supplementary Figure S6.** *In silico* analysis of genetic dependencies in YAP/TAZ high cell lines. (a) Distribution plot of the average Z-score of the YAP/TAZ gene expression signature. Cell lines present in the DepMap database were defined as having high or low YAP/TAZ activity based on the average gene score of a YAP/TAZ gene expression signature. Thresholds were arbitrarily set based on the Z-score distribution. (b) Heatmap displaying the normalized dependency score of genes in YAP/TAZ high or low cell lines. The heatmap reports three classes of genes: gene essential in YAP/TAZ high cells (pink cluster, genes with higher essentiality score (values closer to -1) in YAP/TAZ high cells), gene essential in YAP/TAZ low cells (purple cluster, genes with higher essentiality score in YAP/TAZ low cells) and not-significant genes (green cluster, genes that show the same essentiality score in YAP/TAZ high or YAP/TAZ low cells).

**Supplementary Figure S7.** Essential Pathways in cells with high YAP/TAZ activity. (a) GO-analysis of the genes essential in YAP/TAZ-high cells identified by *in silico* screen of the DepMap database. (b) Word cloud of the most enriched GO terms reported in (a).

Supplementary fig S8

**Supplementary Figure S8.** (a) Kaplan Meyer survival analysis of pan-cancer patients bearing a wild type (red curve) or a missense mutated FERMT2 gene (blue curve). (b) Cell viability of MDA-MB-231 cells treated with the indicated concentration of paclitaxel for 72 hours. Cells were either mock-edited (sgR1) or KO for FERMT2 derived by CRISPR Cas9 editing using two different sgRNAs targeting FERMT2 (sgF1 and sgF2). N=6, ****P value<0.0001. (c) Determination of IC50 for paclitaxel in SUM159PT cells, either mock edited (isgROSA26#2) or KO for FERMT2 derived by CRISPR Cas9 editing using two different sgRNAs targeting FERMT2 (sgFERMT2#1 and #2). N=7, error bar is the stdv. (d) Growth of long-term resistant SUM159PT cells transfected with siRNA against FERMT2 (siFERMT2) or a non-targeting siRNA (siC).

**Supplementary Figure S9.** Waterfall plot of genes reducing the activity of TAZ (luciferase-based reporter). Data are from Sberna et al. (25).

**Supplementary Figure S10.** Expression of YAP/TAZ targets upon silencing of FERMT2 in (a) MCF10A cells and (b) MDA-MB-231 cells. (c) Western blotting analysis of YAP and TAZ expression in MCF10A-Cas9 cells edited with sgRNAs targeting TAZ (sgTAZ) or mock edited with sgROSA26 (sgR26). VNC and GAPDH were used as loading controls.

**Supplementary Figure S11.** Pharmacological inhibition of FAK hampers the expression of YAP/TAZ target genes. (a) Western Blotting analysis to evaluate FAK activity based on its phosphorylation on Tyr397 performed in MDA-MB-231 cells treated with two FAK inhibitors (PND-1186 and PF-431396) at the indicated doses. Mock samples were treated with DMSO. GAPDH was used as loading control.

(b) RT-qPCR analysis of YAP/TAZ targets in MDA-MB-231 cells. n=3, Histogram reports the average and the SD. Two-tailed t-test was applied to determine the statistical significance.

**Supplementary Figure S12.** Levels of actin severing factors assessed by RT-qPCR. MCF10A were silenced with siRNA directed against FERMT2 (#12 and #14), Capzb (siCapzb), Cofilin (siCfl1) or Gelsolin (siGsn). As control, mock silencing was performed with non-targerting siRNAs (siC).

The bar graph reports the average values (n=3) and error bar indicating the stdv. P values were calculated by T-test: ****P<0.00001, ***P<0.0001, **P<0.001, *P<0.01.
